# Cascade-penetrating domino-ferroptosis nano inducer synergizes with sonodynamic therapy for anaplastic thyroid cancer

**DOI:** 10.1016/j.mtbio.2025.102206

**Published:** 2025-08-16

**Authors:** Peng Dong, Yun-Bo Chi, Deng-Ke Teng, Yuan-Qiang Lin, Ling-Yu Zhu, He-Qun Li, Jia-Yu Yang, Jia-Rui Du, Zong-Tao Zhang, Hai-Tao Ran, Guo-Qing Sui, Hui Wang, Qi-Meihui Wang

**Affiliations:** a Department of Ultrasound, China-Japan Union Hospital of Jilin University, No. 126, Xian Tai Street, Changchun, Jilin, 130033, China; b Department of Radiation Oncology, China-Japan Union Hospital of Jilin University, Changchun, Jilin, 130033, China; c State Key Laboratory of Inorganic Synthesis and Preparative Chemistry, College of Chemistry, Jilin University, Changchun, Jilin, 130012, China; d Institute of Ultrasound Imaging, The Second Affiliated Hospital of Chongqing Medical University, Chongqing, 400010, China

**Keywords:** Cascade, Penetrating, Ferroptosis, Sonodynamic therapy, Anaplastic thyroid cancer

## Abstract

Ferroptosis, an iron-dependent, nonapoptotic form of regulated cell death, has become a new approach for antitumor treatment. However, the insufficient accumulation and poor penetration of ferroptosis inducers deep in tumors greatly limit their therapeutic effects. In this study, we constructed a cascade penetrating metal‒polyphenol ultrasonic molecular probe, Fe^3+^Cur-PFP@IR780-LIP (FCIPL). The nanoparticles (NPs) can penetrate deep into tumors in a stepwise fashion via specific targeting combined with cavitation effects resulting from acoustic droplet vaporization (ADV) and ultrasound-targeted microbubble destruction (UTMD) technology. Then, the drug can be successfully delivered into the mitochondria of tumor cells under the cascade response of ultrasound and the tumor microenvironment, inducing the ferroptosis domino cascade. The nanoparticles disassemble, releasing the Fenton reaction catalyst Fe^2+^ and the ferroptosis inducer Cur, which together constitute the ferroptosis "amplifier," promoting the domino-like burst of lipid peroxide (LPO) and triggering ferroptosis. Simultaneously, the sonosensitizer IR780 is activated to induce sonodynamic therapy (SDT). Under the synergistic action of ferroptosis and SDT, a waterfall-like therapeutic effect is produced. In terms of diagnosis, this nanoplatform combines multimodal (ultrasound, photoacoustic, magnetic resonance and fluorescence) imaging to improve the diagnostic performance of anaplastic thyroid cancer (ATC) and provide a visualization strategy for early diagnosis. In this study, the advantages of ultrasound technology are exploited to achieve deep penetration of drugs and overcome the limitations of a single treatment modality, achieve optimal diagnostic and treatment effects, and provide new ideas for integrating ATC diagnosis and treatment.

## Highlights

• Improvement of targeted penetration capabilities and construction of a drug cascade delivery system.
• Provides an innovative strategy for integrated diagnosis and treatment of anaplastic thyroid cancer.
• High therapeutic biosafety.

## 1 Introduction

Improving the clinical efficacy of nanomedicines has long been the focus and challenge of research. The limited penetration and insufficient internal accumulation of nanodrug-carrying systems often lead to unsatisfactory clinical efficacy. The dense extracellular matrix and abundant interstitial cells in tumor tissues limit the penetration of nanoparticles (NPs) into deep tumor tissues [[1], [2], [3]]. Therefore, enhancing the deep penetration ability of nanomaterials is key to achieving efficient treatment. In recent years, phase transformation nanoultrasound contrast agents have been widely studied as ideal ultrasonic molecular probes. Under the action of ultrasound, the probe can undergo acoustic droplet vaporization (ADV) for ultrasound imaging and treatment [4,5]. More importantly, ultrasound-targeted microbubble destruction (UTMD) technology can make it easier for NPs to penetrate the vascular barrier of tumors and enhance the deep tissue penetration, accumulation, and internalization of NPs in tumor tissue in a more controlled manner, effectively improving the delivery efficiency of the contained drugs [6,7]. However, treating tumors via only ADV technology still has the disadvantage of uneven regional treatment effects [8]. Therefore, how to effectively integrate these advantages needs further exploration.

Anaplastic thyroid carcinoma (ATC), a refractory subtype of thyroid malignancy, is highly invasive, malignant and rapidly progressive, with a poor prognosis and a median survival of less than 6 months [[9], [10], [11]]. In terms of diagnosis, there are limitations in its early diagnosis and progression assessment. In terms of treatment, ATC has an insidious onset, and most cases are accompanied by local infiltration, cervical lymph node metastasis and distant metastasis at the time of diagnosis [12]. Once a patient is diagnosed, complete resection by surgical treatment is difficult, radiotherapy and chemotherapy have strong toxic and side effects and drug resistance, and the comprehensive treatment effect is poor [13]. Currently, there is no effective radical treatment method. Therefore, exploring early diagnosis strategies and precise treatment methods has become the key to improving the survival rate of patients with ATC.

As a promising cancer treatment method, sonodynamic therapy (SDT) has the advantages of noninvasiveness, few side effects, strong tissue penetration, and good biological safety [[14], [15], [16]]. Under the action of ultrasound at a certain intensity and frequency, the sonosensitizer is activated to produce reactive oxygen species (ROS) to induce apoptosis in cells [15]. Based on these advantages, the amount of sonosensitizer that accumulates in the tumor area and the selection of the ultrasound parameters determine the antitumor effect of SDT [17]. However, although the use of excellent sonosensitizers is crucial, the resistance of tumor cells to apoptosis, the interstitial pressure within solid tumors, tumor hypoxia, and microenvironments with high concentrations of glutathione (GSH) remain important factors affecting the efficacy of SDT [18,19]. If the treatment is not sufficiently thorough, highly invasive ATC will undoubtedly worsen. Therefore, there is an urgent need to exploit a nonapoptotic mode of cell death to improve the therapeutic efficacy of SDT, overcome the drawbacks of a single treatment modality and eradicate cancer cells.

Ferroptosis is an iron-dependent, nonapoptotic form of cell death. The main characteristics of ferroptosis are increased intracellular iron metabolism and excessive accumulation of lipid peroxides (LPO), which reach lethal concentrations and cause cell death [20]. Considering that iron plays a crucial role in ferroptosis, increasing intracellular iron levels is a powerful approach to induce ferroptosis in tumor cells. Recently, a variety of ferroptosis inducers have been developed for cancer treatment [21,22]. Curcumin (Cur) is an effective polyphenolic compound that induces ferroptosis, and high concentrations of Cur can increase the intracellular iron ion content by increasing the expression of heme oxygenase 1 (HO-1) and further activating the ferroptosis pathway [[23], [24], [25]]. However, despite the excellent therapeutic potential of Cur, its low water solubility and limited biocompatibility limit its efficacy. Fortunately, ions of metals such as Fe, Mn, and Cu can coordinate with the phenolic ligands of polyphenols to form metal‒polyphenol networks (MPNs), which can easily load drugs and release them at tumor sites through the GSH response, greatly increasing the bioavailability of the loaded drugs [[26], [27], [28]]. In addition, iron ions are contrast agents for longitudinal relaxation (T1)-weighted magnetic resonance imaging (MRI) and good catalysts for the Fenton reaction, which causes oxidative stress [29]. Therefore, constructing MPN delivery systems may be an ideal strategy to overcome the difficulties associated with the inclusion of Cur.

Nanomedicine carriers have the advantages of low toxicity, good stability and compatibility, including lipid nanoparticles, polymer carriers, inorganic nanocarrier systems and hybrid nanocarrier systems [30,31]. Liposome nanocarriers have strong biocompatibility and degradability. Compared with polymer carriers and inorganic nanocarrier systems, they can effectively reduce the liver and kidney toxicity of drugs. The lipid carrier structure, similar to the cell membrane, ensures the circulation time of drugs in the body and has a relatively high drug loading efficiency. Hybrid nanocarrier systems are currently being developed rapidly. These systems can integrate the advantages of each component, overcome the shortcomings of single materials, complete multicomponent synergistic therapy, and achieve the integration of diagnosis and treatment [32].

**Scheme 1:**
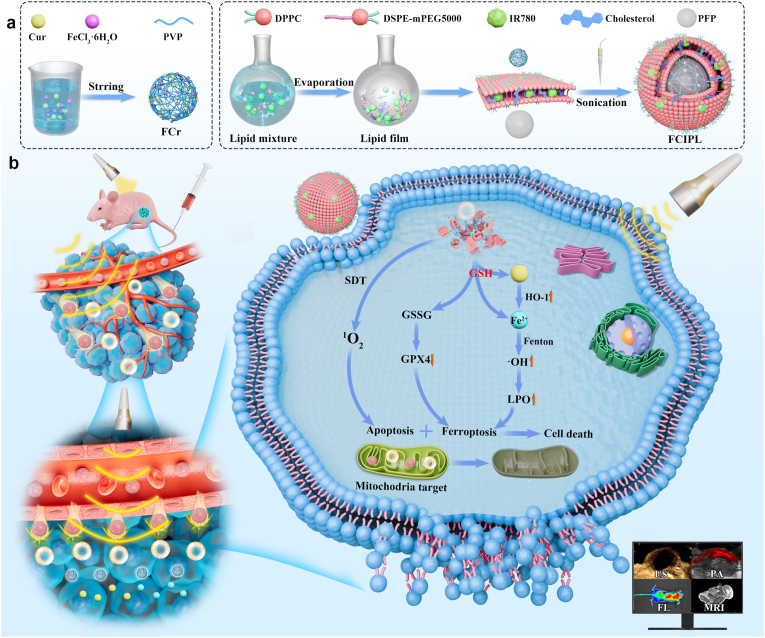
Schematic illustration of the preparation process and therapeutic application of the cascade-penetrating metal‒polyphenol ultrasound molecular probe FCIPL. a) Synthesis process of the FCIPL nanoprobe. b) Schematic illustration of the ability of the cascade-penetrating domino-ferroptosis nano inducer FCIPL to enhance the sonodynamic treatment of ATC. The nanoparticles can penetrate deep into tumor tissue step by step through specific targeting combined with the UTMD caused by ADV, and the drug can be successfully delivered into the mitochondria of tumor cells under the cascade response of ultrasound and the tumor microenvironment, initiating the ferroptosis domino cascade induction system. After disassembly, Fe^2+^ and curcumin constitute a ferroptosis "amplifier" that jointly promotes the domino-effect explosion of LPO, triggers ferroptosis, enhances SDT, and produces a waterfall-like therapeutic effect. In addition, the probe integrates ultrasound, photoacoustic, fluorescence and MRI imaging and has multimodal imaging capabilities.

Herein, we constructed a cascade-penetrating metal–polyphenol ultrasonic molecular probe (Fe^3+^Cur-PFP@IR780-LIP, FCIPL). The FCr core is formed by the self-assembly of the ferroptosis inducers Cur and Fe^3+^, which combine with perfluoropentane (PFP) in liposomes loaded with IR780. IR780 can target the mitochondria of tumor cells, is an excellent sonosensitizer that can induce SDT to cause apoptosis under ultrasound irradiation and can be used as a photoacoustic and fluorescence imaging agent. Moreover, UTMD induced by ADV allows NPs to penetrate deep into tumor tissues and initiate a sequential cascade of drug penetration and release systems. Furthermore, FCr decomposes in the microenvironment due to the high concentration of GSH in tumors, releasing the Fenton reaction catalyst Fe^2+^, which, together with Cur, forms an amplifier for the ferroptosis cascade. This causes a domino effect-like increase in LPOs, triggering ferroptosis, enhancing SDT, and producing a waterfall-like therapeutic effect. In addition, the probe integrates multimodal imaging capabilities (ultrasound, photoacoustic, fluorescence and MRI) (Scheme 1). The nanoprobe constructed in this study activates ferroptosis in deep tumors through targeted penetration and a cascading drug delivery system response, enhancing the therapeutic effect of SDT. Moreover, the nanoprobe can be applied for multimodal molecular imaging and is expected to provide new ideas for combining ATC diagnosis and treatment.

## 2 Results and discussion

### 2.1 Synthesis and characterization of the FCIPL NPs

**Fig. 1:**
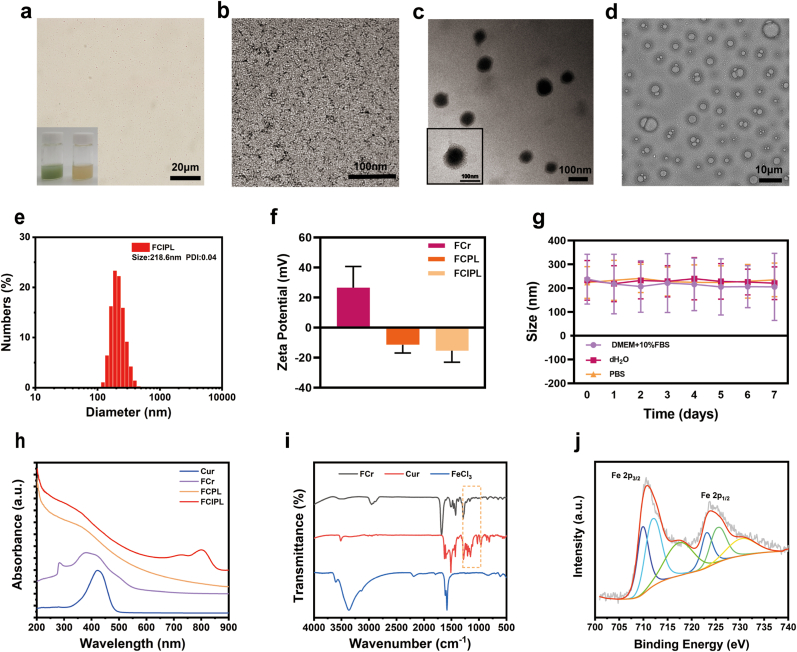
Characterization of FCIPL nanoparticles. a) Optical microscopy image of FCIPL nanoparticles, scale bar is 20 μm. b, c) TEM image of FCr and FCIPL, the scale is 100 nm. d) TEM image of the phase change of FCIPL after Lifu irradiation, the scale is 10 μm. e) Particle size distribution of FCIPL. f) Potential values of FCr, FCPL and FCIPL. g) The 7-day stability of FCIPL. h) UV–visible absorption spectra of Cur, FCr, FCPL and FCIPL. i) Infrared spectra of FeCl_3_, Cur and FCr. j) Fe element XPS fitting curve of FCIPL.

This study synthesized nanoparticles FCIPL using the self-assembly method and the vacuum film-sonic shock method [33,34]. As shown in the transmission electron microscopy (TEM) and light microscopy images, FCIPL had a regular spherical morphology and exhibited excellent uniformity and dispersion (Fig. 1a and c), whereas FCr appeared as extremely small point-like uniformly distributed NPs (Fig. 1b). After low-intensity focused ultrasound (Lifu) irradiation, the FCIPL NPs undergo a liquid‒gas phase transition and appear as microbubbles of varying sizes (Fig. 1d). The favorable ADV properties of the FCIPL are a prerequisite for its use in ultrasound molecular imaging applications and deep drug penetration. The solution of the prepared FCIPL NPs was grass green, whereas the FCPL solution was light brown (S1), confirming the effective loading of IR780. The average hydrated particle sizes of FCIPL, FCPL and FCr measured via dynamic light scattering (DLS) were 218.6 ± 55.77 nm, 202.3 ± 100.2 nm, and 15.33 ± 2.34 nm, respectively, and the zeta potentials were −15.4 ± 7.66 mV, −11.5 ± 5.41 mV, and 26.6 ± 14.1 mV, respectively (Fig. 1e and f, S2, S3, S4). Owing to its particle size, FCIPL can effectively reach the tumor site through the gap between vascular endothelial cells [[35], [36], [37], [38]]. Moreover, the slightly negative potential of these NPs ensures their stable dispersion in the blood circulation, effectively preventing aggregation [39]. The increase in NPs’ size indirectly indicated that IR780 was successfully loaded. The average particle size of the FCIPL NPs remained relatively stable over 7 days (Fig. 1g). These results indicated that the FCIPL NPs have desirable long-term stability.

**Fig. 2:**
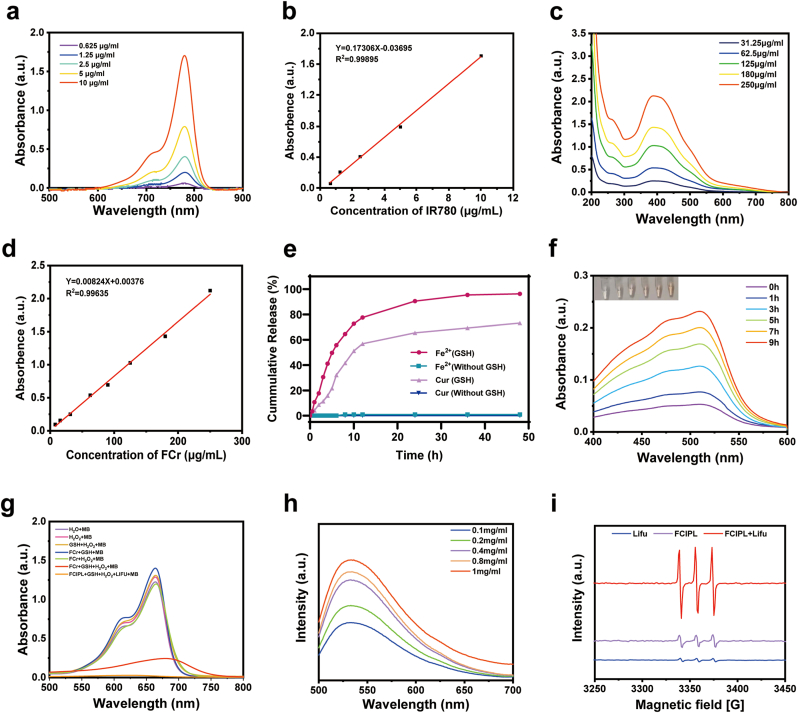
Basic properties of FCIPL. a) UV absorption spectrum of IR780 at different concentrations. b) Corresponding concentration‒absorption intensity relationship of IR780. c) UV absorption spectrum of FCr at different concentrations. d) Corresponding concentration‒absorption intensity relationship of FCr. e) Accumulative curcumin and Fe^2+^ release of FCIPL with and without GSH. f) UV–visible absorption spectrum of Fe^2+^ released by FCIPL in GSH solution at different times. g) MB detection of the production of ·OH. h) SOSG detection of the production of ^1^O_2_ by FCIPL at different concentrations after Lifu irradiation. i) ESR spectra of ^1^O_2_ produced by the reaction system under different treatment conditions with TEMP as the trapping agent.

The UV absorption spectrum of the aqueous solution of FCr (Fig. 1h) shows a characteristic absorption peak at 380 nm, which is different from that of Cur and indirectly indicates successful coordination between Fe^3+^ and Cur. Compared with that of FCPL, the UV spectrum of FCIPL contains a unique absorption peak from IR780 at approximately 800 nm, confirming the successful loading of IR780. The redshift in the absorbance peak could be attributed to solvent effects or intermolecular interactions [40]. In addition, Fig. 2a–d shows the linear relationship between the concentrations of IR780 and FCr and their absorbance values (R_IR780_^2^ = 0.999, R_FCr_^2^ = 0.996). The encapsulation efficiency (EE) and loading capacity (LC) of IR780 in FCIPL were 91.86 % and 4.37 %, respectively, whereas the EE and LC of FCr were 47.06 % and 1.92 %, respectively. The infrared spectrum of FCr showed a decrease in the intensity of the peak at 1150–1200 cm^−1^ (HO-C stretching band), indicating the successful coordination of the metal polyphenols (Fig. 1i). The valence state of Fe in FCIPL was analyzed by X-ray photoelectron spectroscopy (XPS), and the Fe 2p_2/3_ and Fe 2p_1/2_ binding energy peaks at 710.1 eV and 723.7 eV, respectively (Fig. 1j).

### 2.2 GSH consumption and ROS production by FCIPL

High GSH concentrations, slight acidity and high H_2_O_2_ concentrations are characteristic of the tumor microenvironment [41]; therefore, we examined the in vitro drug release from FCIPL and its GSH consumption capacity. O-Phenanthroline reacted with Fe^2+^ to form stable orange‒red o-diazophenanthrene ferrous ions with a UV absorption peak at 510 nm. As shown in Fig. 2f, after the addition of o-phenanthroline, the solution changed from colorless to orange‒red with increasing time, and the intensity of the characteristic UV absorption peak at 510 nm gradually increased. Similarly, 5,5′-dithiobis (2-nitrobenzoic acid) (DTNB) can be used as an indicator probe to generate a colored product in the presence of (unconsumed) GSH, with an absorbance maximum at 412 nm. Notably, the characteristic UV absorption peak at 412 nm tended to decrease over time with the addition of DTNB (Fig. S6). We included the Cur-PFP@IR780-LIP group as a negative control, which showed no 10.13039/501100022272GSH depletion or Fe^2+^ generation (Supporting document Figs. S5 and S7), thereby excluding the influence of other components on 10.13039/501100022272GSH levels. Upon monitoring by ICP-AES at different time points, as high as 96.27 % of Fe^2+^ can be released after 48h incubation (Fig. 2e). In contrast, there is not any detectable Fe^2+^ released from FCIPL in the absence of GSH. Moreover, the release curves of curcumin with or without GSH are exhibited in Fig. 2e, almost 73.14 % of curcumin can be released from FCIPL after incubation with GSH for 48 h. These findings demonstrated that FCIPL could disassemble in the presence of high concentrations of GSH, consume GSH, and be reduced to release Fe^2+^ and curcumin, providing an environment with iron overload for ferroptosis to occur.

To confirm the ability of this nanomaterial to generate ROS, we measured the contents of hydroxyl radicals (·OH) and singlet oxygen (^1^O_2_). Methylene blue (MB) can be oxidized by ·OH; therefore, MB was used as a probe to detect ·OH. As shown in Fig. 2g, the maximum UV absorption of the MB solution decreased significantly in the FCr and FCIPL + Lifu groups, and the solution changed from blue to colorless after sequential treatment with GSH and H_2_O_2_. This change was more obvious and occurred faster in the FCIPL + Lifu group, confirming that FCr can disassemble in the presence of high concentrations of GSH to promote the Fenton reaction and produce ·OH and that ultrasound can accelerate this reaction. The principle may be that the FCIPL undergoes ADV after Lifu irradiation, and the resulting microbubbles fuse and rupture due to cavitation and UTMD, which significantly promotes drug release.

To evaluate the SDT performance of FCIPL, 1,3-diphenylisobenzofuran (DPBF) and singlet oxygen sensor green (SOSG) were used as probes to detect the ^1^O_2_ produced by Lifu irradiation. DPBF is a fluorescent probe whose fluorescence is quenched after combining with singlet oxygen, and its absorption intensity under UV–visible light (465 nm) also decreases rapidly. Fig. S8 shows that with increasing ultrasonic irradiation time, the UV absorption peak tended to decrease, indicating that FCIPL has a strong ability to produce ^1^O_2_ under ultrasonic irradiation. Similarly, SOSG is highly selective for ^1^O_2_ and emits green fluorescence upon binding to this molecule, with excitation/emission wavelengths of 504 nm/525 nm. As shown in Fig. 2h, the fluorescence intensity at 525 nm increased with increasing FCIPL concentration; that is, more ^1^O_2_ was produced. In addition, the SDT effect of FCIPL was verified via electron spin resonance (ESR), where the signal of ^1^O_2_ was monitored via the trapping agent TEMP. Compared with the Lifu and FCIPL groups, the FCIPL + Lifu group presented highly characteristic (1:1:1) signal from ^1^O_2_ (Fig. 2i); thus, these data are consistent with the above results.

### 2.3 In vitro multimodal imaging with FCIPL

**Fig. 3:**
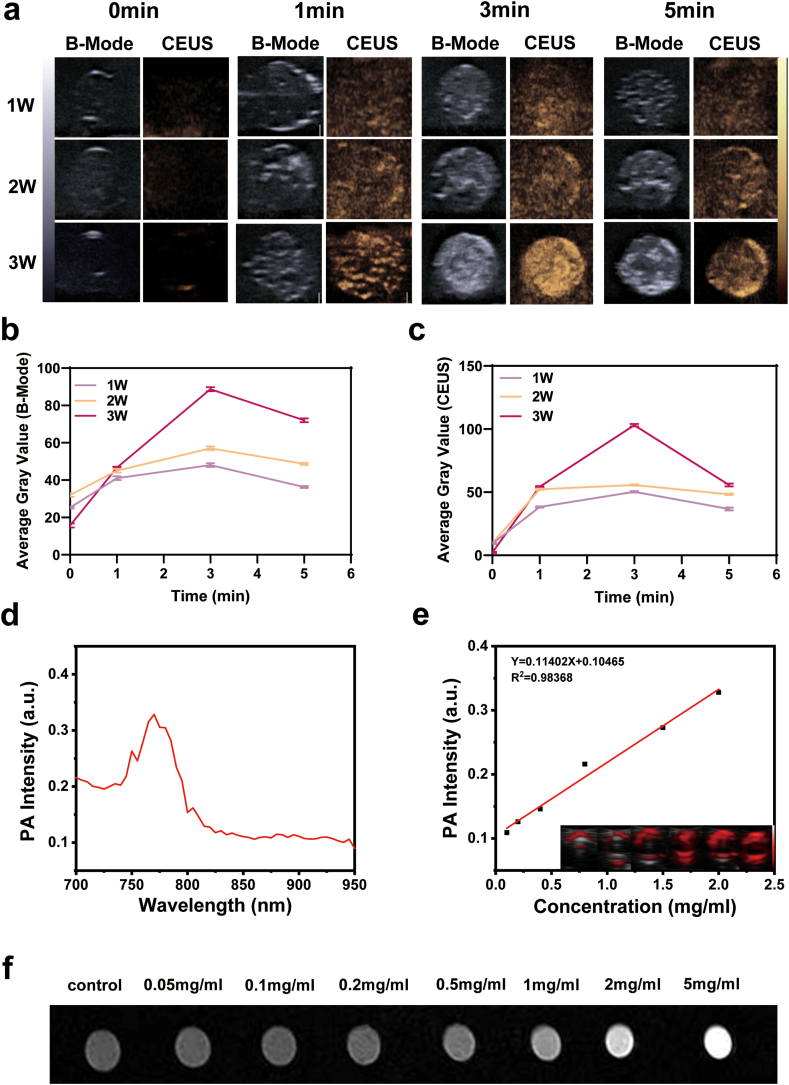
In vitro multimodal imaging. a) US images of FCIPL NPs after Lifu irradiation with different irradiation intensities at different time points. b, c) Relative gray values of B-Mode and CEUS images after Lifu irradiation at different time points (n = 3). d) Changes in the PA signal in the FCIPL in the wavelength range of 700–950 nm. e) Quantitative analysis of the relationship between the FCIPL concentration and PA signal intensity. Inset: PA image of FCIPL at increasing concentrations. f) In vitro MR images of different concentrations of FCIPL.

Because FCIPL has good ADV capability, we evaluated the ultrasound imaging performance of the FCIPL NPs. Compared with that of the phosphate-buffered saline (PBS) group, the gray value of the FCIPL group increased significantly after ultrasound irradiation (Figs. S9 and S10), and the gray values of the ultrasound images in both 2D mode and contrast-enhanced ultrasound (CEUS) mode increased with increasing irradiation intensity. The gray value was the highest and peaked at 3 min (3 W), after which it decreased for 5 min. This may be due to the gradual increase in and rupture of the microbubbles produced by ADV over time (Fig. 3a, b, 3c). The above results confirmed that FCIPL has good ultrasound imaging capabilities and can be used as an ultrasound contrast agent.

Photoacoustic imaging effectively combines the advantages of optical imaging (high sensitivity) and ultrasound imaging (high resolution), which is important for efficacy evaluations. The photoacoustic curve generated from the full-wavelength scan (700–950 nm) of the photoacoustic imaging system (Fig. 3d) showed a peak at 770 nm for FCIPL. Therefore, 770 nm was chosen as the photoacoustic irradiation wavelength, and the photoacoustic signal gradually and linearly increased with increasing NPs concentration (R^2^ = 0.9837) (Fig. 3e), indicating that FCIPL has good photoacoustic imaging capability.

Transition metals such as Fe, Mn and Cu can be used for T1 MR imaging. As shown in Fig. 3f, compared with that of PBS, the MRI applicability of FCIPL NPs was greater, and the MRI signal was enhanced with increasing FCIPL concentration. IR780 is a good fluorescence developer, and the IR780 fluorescence intensity of the FCIPL group was greater than that of the control group, indicating that FCIPL can be used to perform fluorescence imaging and as a fluorescence imaging developer (Fig. S11).

Ultrasound molecular imaging has the advantages of safety, simplicity, real-time dynamics, deep penetration, and strong signal amplification. Fluorescence imaging has extremely high sensitivity and specificity [42]. Photoacoustic imaging combines the strong sensitivity of optical imaging and the high spatial resolution of ultrasound imaging. It can also assess the microvascular volume in tumor tissues by detecting oxygen and hemoglobin levels, which is of great significance for evaluating therapeutic efficacy and is an important supplement to ultrasound molecular imaging [43]. For the invasive characteristics of ATC, MRI can analyze the invasion of the lesion to surrounding tissues and the involvement of lymph nodes and distant metastases, thereby improving the shortcomings of other examination methods that are prone to missed diagnoses [44]. Therefore, combining ultrasound imaging, MRI, photoacoustic imaging, and fluorescence imaging, integrating their respective advantages, and fully exerting the diagnostic efficacy of multimodal molecular imaging, provides a new strategy for visualized and precise diagnosis and treatment of ATC.

### 2.4 In vitro uptake and targeting

**Fig. 4:**
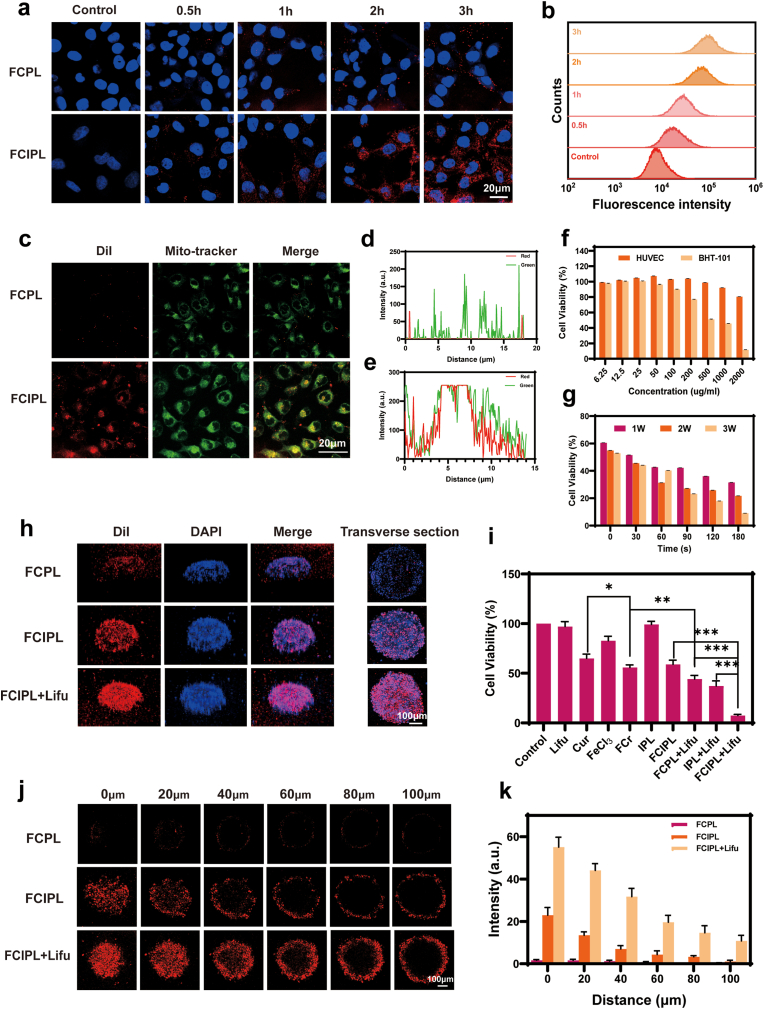
In vitro targeting, cytotoxicity and penetration of FCIPL NPs. a) Uptake of FCPL (1st row) and FCIPL (2nd row) by BHT-101 cells at different time points, scale bar is 20 μm. b) FCM quantification of the ability of BHT-101 cells to uptake FCIPL nanoparticles at different incubation times. c) Organelle localization of FCPL, FCIPL in BHT-101 cells, from left to right, the order of staining is as follows: DiI-labeled nanoparticles, MitoTracker-labeled mitochondria, fusion image, the scale bar represents 20 μm. d,e) FCPL and FCIPL's mitochondria co-localization efficiency was measured using a line-scan profile. f) Biosafety and cytotoxicity of FCIPL nanoparticles. g) Cytotoxicity of FCIPL nanoparticles treated with different durations and concentrations of Lifu. h) 3D images of FCPL, FCIPL, and FCIPL + Lifu in the 3D tumor ball and corresponding cross-sectional images. i) Effects of different treatments on cell activity (n = 4, ∗P < 0.05, ∗∗P < 0.01, ∗∗∗P < 0.001). j, k) Penetration images and quantitative analysis of fluorescence intensity of FCPL, FCIPL, and FCIPL + Lifu in different layers of the 3D tumor ball, scale bar is 100 μm.

To ensure that the nanomaterials are accurately localized in the tumor location for early disease diagnosis and targeted treatment, we chose the specific acoustic sensitizer IR780 because of its excellent tumor mitochondria-targeting effects [45]. The NPs were labeled with 1,1′-dioctadecyl-3,3,3′,3′-tetramethyl indocarbocyanine perchlorate (DiI), which produces red fluorescence that can be accurately identified via confocal laser scanning microscopy (CLSM). As shown in Fig. 4a, the amount of NPs ingested by the cells increased gradually with increasing incubation time, the red fluorescence in the cells was the strongest, and the best phagocytosis effect was achieved at 3 h. Moreover, at the same time, the BHT-101 cells ingested much more DiI-FCIPL than DiI-FCPL, confirming the targeting effect of IR780. The cellular uptake rates after different incubation durations (0.5, 1, 2, and 3 h), as determined by flow cytometry (FCM), were 21.01 %, 50.55 %, 96.57 %, and 99.24 %, respectively (Fig. 4b). The cells had essentially taken up all the NPs after 3 h; therefore, we chose 3 h as the incubation time for the subsequent experiments.

To further explore the ability of the FCIPL NPs to target certain organelles, we used MitoTracker Green to perform colocalization experiments and stain the mitochondria of BHT-101 cells, as shown in Fig. 4c, d, 4e. The red fluorescence of DiI-FCIPL colocalized with the green fluorescence of MitoTracker Green, resulting in strong yellow fluorescence signals, whereas the DiI-FCPL group presented almost no yellow fluorescence signal. Therefore, FCIPL can accumulate and act in the mitochondria of BHT-101 cells. The targeted molecule IR780 is used for mitochondrial targeting in tumor cells. The subcellular localization of nanomaterials can significantly affect the therapeutic efficacy of tumors [46]. Due to the inherent lipophilicity and overall positive charge of IR780, and the negative potential of the mitochondrial membrane, tumor cell mitochondria are more active and have a higher potential compared to normal tissues. The attraction between cations and anions facilitates the accumulation of IR780-loaded nanoparticles in mitochondria. Additionally, tumor cells preferentially generate energy through the glycolytic pathway, and IR780 possesses cationic transporter peptides OATPS, which are increasingly expressed in tumor cells, thereby promoting the aggregation of IR780 in tumor cells [47].

### 2.5 Deep penetration of the FCIPL NPs in vitro

The deep penetration of drug-carrying nanosystems into tumor tissues has always been a major challenge in nanomaterial applications, and the heterogeneity of the tumor microenvironment and the dense extracellular matrix of tumor tissues are the main obstacles to NPs enrichment inside tumors [1,3]. Ultrasound is considered a good stimulation method to promote deep drug penetration into tumors and has been demonstrated in several studies [7]. Therefore, we simulated the tumor microenvironment by constructing a 3D tumor sphere model to evaluate the penetration ability of FCIPL. We found that when there was no IR780 in the NPs, there was almost no NP aggregation in the tumor sphere. Moreover, in the absence of Lifu irradiation, most of the DiI-FCIPL NPs aggregated on the surface of the tumor sphere, whereas upon Lifu irradiation, many DiI-FCIPL NPs penetrated through the tumor sphere and into deeper tissues and were uniformly distributed in the core of the model (Fig. 4h, j, 4k). The above results confirmed the targeting of the FCIPL NPs and the role that ultrasound plays in promoting deep drug penetration. In addition, ultrasound was confirmed to have sufficient penetration depth, as verified in Figs. S12 and S13.

The targeting molecule IR780 can be involved in both deep tumor penetration and mitochondrial targeting of tumor cells. With the assistance of IR780, nanoparticles penetrate deeply into tumor tissues after blood circulation, which effectively improves tumor permeability and allows nanoparticles to penetrate deeply into tumor tissues [48,49]. In addition, the subcellular localization of nanomaterials can significantly affect the therapeutic effect of tumors [46].

### 2.6 In vitro cytotoxicity

The safety and cytotoxicity of different concentrations of FCIPL NPs were investigated using the cell counting kit-8 (CCK-8) method. As shown in Fig. 4f, the cellular activities of HUVECs and BHT-101 cells gradually decreased with increasing NPs concentration, although the HUVEC survival rate was greater than 95 % at NPs concentrations not greater than 1000 μg/mL, which indicated that in the absence of ultrasound irradiation, the FCIPL NPs were not obviously toxic to normal cells and had good biocompatibility. However, the BHT-101 cell survival rate was 45.24 % ± 1.13 % when the FCIPL NPs concentration was 1000 μg/mL; therefore, we used 1000 μg/mL NPs for further experiments, as this concentration can kill tumor cells while protecting normal cells. Moreover, we screened the applicable Lifu parameters for treatment, as shown in Fig. 4g. As the Lifu irradiation intensity and irradiation time increased, the cell survival rate gradually decreased, and Lifu irradiation at 3 W for 3 min resulted in a cell survival rate of only 9 % ± 2.77 %.

**Fig. 5:**
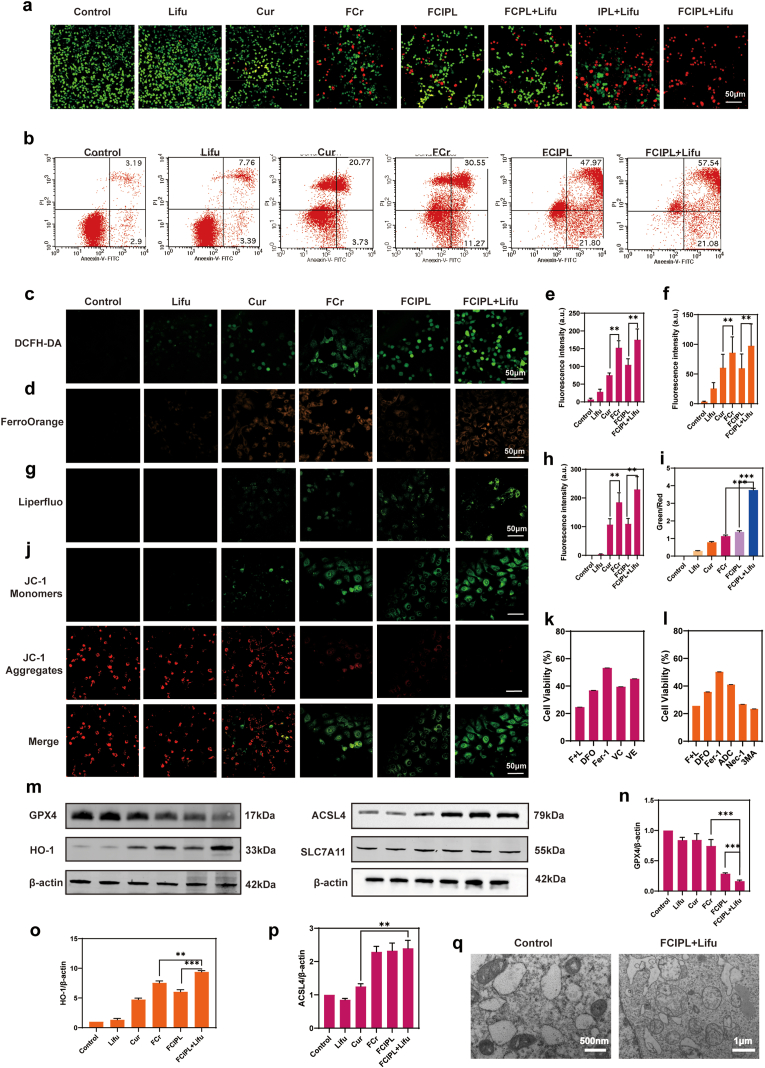
Cytotoxicity mechanism of FCIPL and the synergistic therapeutic effect of sonodynamic therapy combined with ferroptosis. a) CLSM images of live‒dead staining of BHT-101 cells with different treatments (CAM staining in green for live cells and PI staining in red for dead cells), the scale bar is 50 μm. b) FCM analysis of apoptosis in BHT-101 cells after different treatments, the numbers represent the percentage of apoptotic cells. c, e) Confocal images of ROS generated by BHT-101 cells after different treatments and quantitative analysis of fluorescence intensity, the scale bar is 50 μm. d,f) Fe^2+^ production in BHT-101 cells after different treatments and quantitative analysis of fluorescence intensity, the scale bar is 50 μm. g, h) LPO production in BHT-101 cells after different treatments and quantitative analysis of fluorescence intensity; the scale bar represents 50 μm. j, i) Changes in the mitochondrial membrane potential of BHT-101 cells after different treatments and quantitative analysis of fluorescence intensity, ; the scale bar represents 50 μm. k) Effects of ferroptosis inhibitors on cell survival. l) Effects of other inhibitors on cell survival. m, n, o, p) Effects of different treatments on protein expression in BHT-101 cells and quantitative analysis. q) TEM images of BHT-101 cells subjected to different treatments.

In recent years, some studies have combined ferroptosis with other therapeutic modalities and obtained good results [13,50,51]. To verify the synergistic therapeutic effect of SDT and ferroptosis in this study, we used different methods to treat BHT-101 cells and observed changes in their survival rates. As shown in Figs. S14 and 4i, compared with that of the control group, the cell survival rate of the Lifu-treated group was 97.06 % ± 4.95 %, which was essentially the same as that of the control group, indicating that Lifu had no effect on the cell survival rate. The cell survival rates of the FeCl_3_ group and the IPL group were 82.85 % ± 4.48 % and 99.23 % ± 3.08 %, respectively. Additionally, the cell survival rates of the Cur- and FCr-treated groups were 64.92 % ± 4.43 % and 55.79 % ± 2.61 %, respectively, demonstrating that our metal–polyphenol complexes were more cytotoxic and promoted ferroptosis better than pure Cur did. At high concentrations (≥20 μM), Cur can act as an inducer of ferroptosis in tumor cells [52]. Thus, it is essential that FCr disassembles to produce sufficient Cur to induce ferroptosis, and the high GSH concentration in the tumor microenvironment provides sufficient conditions for this purpose. In addition, the cell survival rate of the FCPL + Lifu group was 44.15 % ± 3.63 %, indicating that this treatment had a stronger therapeutic effect than did FCr, confirming that the NPs encapsulating PFP prompted ADV under ultrasound irradiation and that UTMD led to an increase in the number of NPs entering the cells, which therefore increased toxicity. Moreover, the cytotoxicity in the FCIPL + Lifu group was significantly greater than that in the FCIPL group, with cell survival rates of 7.46 % ± 1.11 % and 58.89 % ± 4.28 %, respectively, indicating that Lifu can stimulate SDT to significantly improve the therapeutic effect of FCIPL. Furthermore, the cell survival rate of the IPL + Lifu group was 37.18 % ± 5.13 %, indicating that FCIPL + Lifu had a greater killing effect and confirming that ferroptosis can effectively enhance SDT and achieve synergistic effects. Similarly, FCIPL + Lifu had stronger cytotoxicity than did FCPL + Lifu, which indirectly confirmed the targeting effect of IR780 and the efficacy of the acoustic sensitizers. A comparison of the Cur, FCr, FCPL + Lifu, and FCIPL + Lifu groups revealed that a cascade response and sequential drug release from the NPs occurred. Under ultrasound irradiation, the NPs underwent ADV, released FCr via UTMD while simultaneously stimulating SDT, and then further disassembly of FCr released Cur and Fe^2+^ to induce ferroptosis, successfully achieving ferroptosis-enhanced SDT. The CLSM results after live–dead cell staining were consistent with the above findings, and after FCIPL + Lifu treatment, essentially all the cells were dead (Fig. 5a). Notably, similar results in the apoptosis assay were observed by FCM (Fig. 5b), and the cell-damaging FCIPL + Lifu group presented the highest percentage of apoptotic cells (78.62 %).

### 2.7 Ferroptosis-augmented SDT mechanisms of FCIPL in vitro

We next explored the cytotoxic mechanism of action of FCIPL by adding different inhibitors (Fig. 5k and l). The cells treated with each of the four ferroptosis inhibitors (deferoxamine (DFO), ferrostatin-1 (Fer-1), vitamin C (VC), and vitamin E (VE)) presented higher survival rates than the FCIPL + Lifu-treated cells did. The cell survival rate of the FCIPL + Lifu group was 24.66 % ± 4.86 %, whereas the survival rates of the DFO, Fer-1, VC and VE groups were 36.83 ± 7.36 %, 53.23 % ± 8.89 %, and 39.47 % ± 1.22 %, respectively. 45.29 % ± 8.97 %, respectively. The effect of Fer-1 was the most notable, with a 115.86 % increase in cell survival, confirming that the cell injury caused by the nanoplatform was related to ferroptosis with a mechanism involving intracellular iron overload and LPO accumulation. In addition, the apoptosis inhibitor Ac-DEVD-CHO (APO) significantly increased cell survival (41.03 % ± 7.05 %), whereas necrostatin-1 (Nec-1; a necrosis inhibitor) and 3-methyladenine (3 MA; an autophagy inhibitor) did not prevent cell death. Because the addition of ADC improved cell survival, this finding also indirectly indicates that the mechanism of FCIPL-induced cellular damage is closely related to apoptosis caused by SDT.

2′,7′-Dichlorofluorescein diacetate (DCFH-DA) is a fluorescent ROS indicator probe that can permeate the cell membrane. After entering the cell, DCFH-DA can be oxidized by intracellular ROS to produce the green, strongly fluorescent substance 2′,7′-dichlorofluorescein; thus, intracellular ROS can be detected according to the change in DCFH-DA fluorescence intensity [53]. Our CLSM observations of the ROS contents in cells subjected to different treatments revealed that the fluorescence intensities tended to gradually increase, with the strongest fluorescence intensity in the FCIPL + Lifu group (Fig. 5c,e). These data were verified via FCM, and the percentages of ROS generated in the control, Lifu, Cur, FCr, FCIPL, and FCIPL + Lifu groups were 0.56 %, 34.9 %, 67.7 %, 78.3 %, 82.7 %, and 97.4 %, respectively (Fig. S15).

Characteristic hallmarks of ferroptosis are increased levels of Fe^2+^, depletion of GSH, downregulation of glutathione peroxidase 4 (GPX4), and accumulation of LPOs [54]. Because the fluorescence intensity of FerroOrange is proportional to the concentration of Fe^2+^, we used FerroOrange to determine the intracellular levels of Fe^2+^. As shown in Fig. 5d,f, the orange fluorescence intensity of the FCr group was slightly stronger than that of the Cur group, suggesting that the constituent metal–polyphenol complexes had disassembled and been reduced to Fe^2+^ in BHT-101 cells and that the intracellular content of Fe^2+^ had increased. Moreover, the fluorescence intensities of the control and Lifu groups were almost zero, whereas the fluorescence intensity of the FCIPL + Lifu group was greater than that of the FCIPL group. These data prove that the NPs could disassemble and release Fe^2+^ after phase transition and bursting under Lifu irradiation, whereas Lifu alone had no effect on the intracellular Fe^2+^ content.

The accumulation of large amounts of ROS in the cell can lead to mitochondrial membrane depolarization and subsequent apoptosis. Notably, alterations in the mitochondrial membrane potential can be detected by the fluorescent probe JC-1. When the membrane potential changes, JC-1 monomers become JC-1 aggregates, and the probe emits green and red fluorescence, indicating low-potential damaged mitochondria and high-potential normal mitochondria, respectively, as shown in Fig. 5j and i. Stimulation with Lifu alone has essentially no effect on the mitochondrial membrane potential. Upon the addition of Cur or FCr, the cells underwent ferroptosis, the mitochondrial membrane potential decreased, and the intensity of green fluorescence in the FCr group was greater than that in the Cur group, indicating that the ferroptosis effect of FCr was stronger. The mitochondrial membrane potential in the FCIPL + Lifu group was significantly lower than that in the FCr group, as evidenced by a significant increase in the green/red fluorescence ratio in the CLSM images, suggesting that, in the presence of Lifu irradiation, cellular ferroptosis could potentiate SDT.

To detect intracellular LPO production, we used the LPO probe Liperfluo, which is specifically oxidized by lipid peroxides and emits green fluorescence. CLSM revealed almost no green fluorescence in the Lifu group and only weak green fluorescence in the Cur and FCIPL groups. The enhanced fluorescence in the FCr group compared with that in the Cur group indicated that the ferroptosis effect of FCr was stronger than that of Cur, as it can cause lipid peroxidation to generate more LPOs. Importantly, the strongest fluorescence intensity was observed in the FCIPL + Lifu group, demonstrating that under the simultaneous effects of SDT and ferroptosis, large amounts of LPOs can be generated to act on the cells, resulting in cell death (Fig. 5g and h).

Western blot experiments revealed that FCIPL + Lifu significantly downregulated the protein expression of GPX4 and significantly increased the protein expression of HO-1. This result also verified our hypothesis that FCIPL released FCr under ultrasound irradiation and further disassembled to release Cur in tumor cells. Cur increased the Fe^2+^ content by upregulating HO-1 in tumor cells, which promoted the Fenton reaction and GSH depletion, thereby downregulating GPX4 and generating large amounts of LPOs, causing cellular ferroptosis (Fig. 5m, n, 5o). Furthermore, we performed Western blot analysis of ACSL4 and SLC7A11 proteins (Fig. 5m and p). The results showed that under the stimulation of FCIPL + Lifu, the expression level of ACSL4 protein significantly increased, while the expression level of SLC7A11 protein remained largely unchanged. ACSL4 can activate long-chain polyunsaturated fatty acids (PUFA) and integrate them into membrane phospholipids (such as PE-PUFA), providing substrates for lipid peroxidation [55]. Under ultrasound stimulation, FCIPL induces ferroptosis, leading to the downregulation of GPX4 protein and the accumulation of lipid peroxidation. Cells sense oxidative damage and, in order to repair the damaged cell membrane, further upregulate ACSL4 through stress signals, accelerating the synthesis of PUFA phospholipids. However, this process instead provides more substrates for peroxidation, promoting the occurrence of ferroptosis in cells.

To study the specific morphological changes caused by ferroptosis in cells, we used TEM to observe FCIPL + Lifu-treated BHT-101 cells (Fig. 5q). Compared with the PBS-treated cells, the FCIPL + Lifu-treated cells had fewer total mitochondria; these mitochondria displayed fracture, lysis, and disappearance of the mitochondrial ridges; and the density of the mitochondrial membranes increased. These microscopic morphological changes are consistent with the characteristics of ferroptosis.

**Fig. 6:**
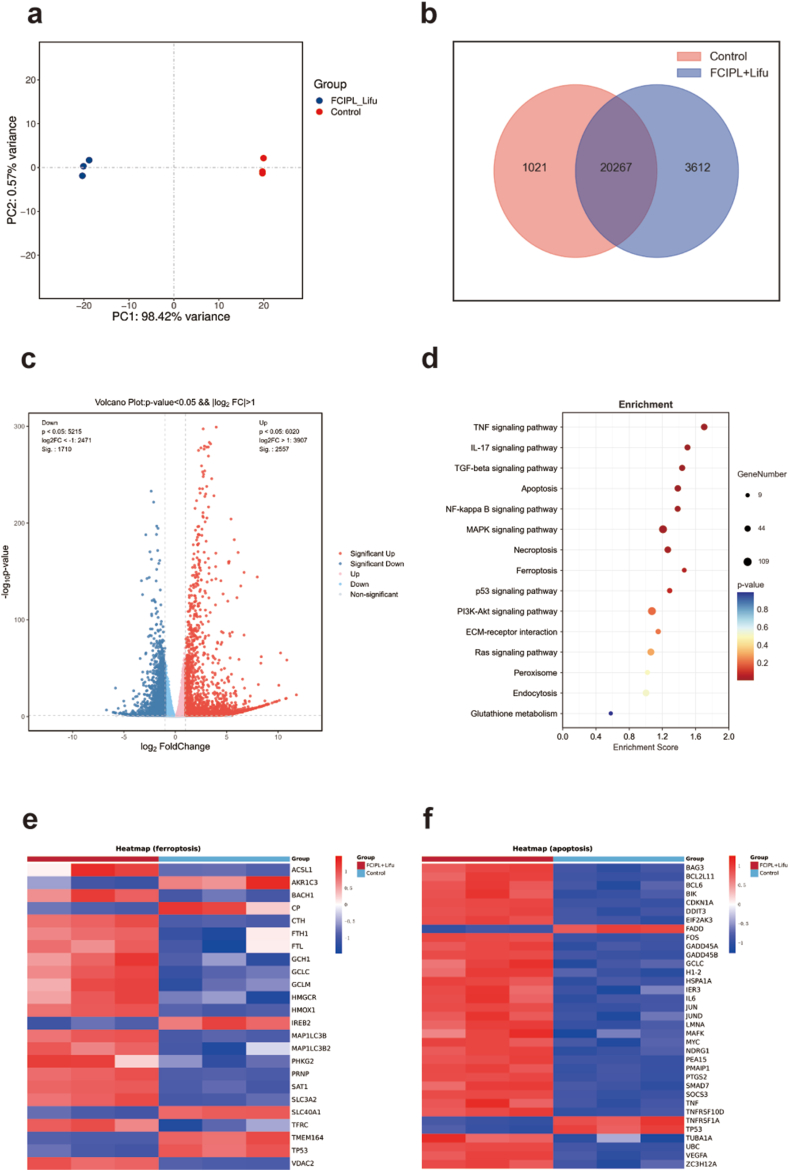
Mechanism of FCIPL under Lifu irradiation according to RNA sequencing. a) Principal component analysis. b) Venn diagram of the differentially expressed genes in the control and FCIPL + Lifu groups. c) Volcano plot of the differentially expressed genes in the control and FCIPL + Lifu groups. d) Kyoto Encyclopedia of Genes and Genomes (KEGG) enrichment analysis showing the differentially expressed genes between the control and FCIPL + Lifu groups. e, f) Heatmap of genes that were differentially expressed in the BHT-101 cells treated with FCIPL + Lifu compared with the control cells (fold change [FC] ≥ 1.5 [or −1.5], *p* < 0.05).

To further explore the potential mechanism of ferroptosis-enhanced SDT by FCIPL + Lifu, after incubation with PBS or FCIPL + Lifu for 24 h, two groups of treated BHT-101 cells were collected for transcriptomic analysis. Principal component analysis and heatmaps revealed significant differences in gene expression between the control and FCIPL + Lifu groups (Fig. 6a, Fig. S16). As shown in the Venn diagram, the two groups of samples expressed a total of 20,267 genes, whereas the FCIPL + Lifu group alone expressed a total of 3612 genes (Fig. 6b). The volcano plot revealed a significant difference in gene expression between the two groups, indicating that FCIPL + Lifu had a significant impact on the molecular biological mechanism of BHT-101 cells (Fig. 6c). To determine the pathways underlying FCIPL + Lifu-induced ferroptosis and SDT, we performed Kyoto Encyclopedia of Genes and Genomes (KEGG) analysis to identify enriched gene sets, and multiple cellular pathways, including the mitogen-activated protein kinase (MAPK), apoptosis, ferroptosis, and glutathione metabolism signaling pathways, were significantly increased after FCIPL + Lifu treatment (Fig. 6d). The ferroptosis-related genes TFRC, HO-1, etc., were significantly differentially expressed (Fig. 6e). In addition, FCIPL + Lifu treatment significantly upregulated or downregulated the expression of apoptosis-related genes, which are closely related to apoptosis, in tumor cells (Fig. 6f). In summary, under Lifu irradiation, FCIPL can actively induce apoptosis and ferroptosis in BHT-101 cells, effectively killing cancer cells.

### 2.8 In vivo biosafety

Before the tumor treatment experiments, we examined the biosafety of FCIPL. Blood samples were collected from the mice injected with the FCIPL NPs on days 1, 3, 7, 15, and 30 and were compared with those from the control group. There were no significant differences in the blood indices (red blood cells (RBCs), white blood cells (WBCs), lymphocytes (Lymphs), platelets (PLTs), hemoglobin (HCG), hematocrit (HCT), mean corpuscular volume (MCV), and mean corpuscular hemoglobin concentration (MCHC)), liver function indices (ALT and AST), kidney function indices (CREA and UREA), or cardiac enzymes (CK) among the groups (Fig. S17). Compared with those in the control group, no acute or chronic histological changes were observed in the hematoxylin and eosin (H&E)-stained major organs at any time point in the FCIPL group (Fig. S18). These experimental results showed that FCIPL NPs have good safety and biocompatibility, which is a prerequisite for their application in in vivo experiments.

### 2.9 In vivo targeting and multimodal imaging

**Fig. 7:**
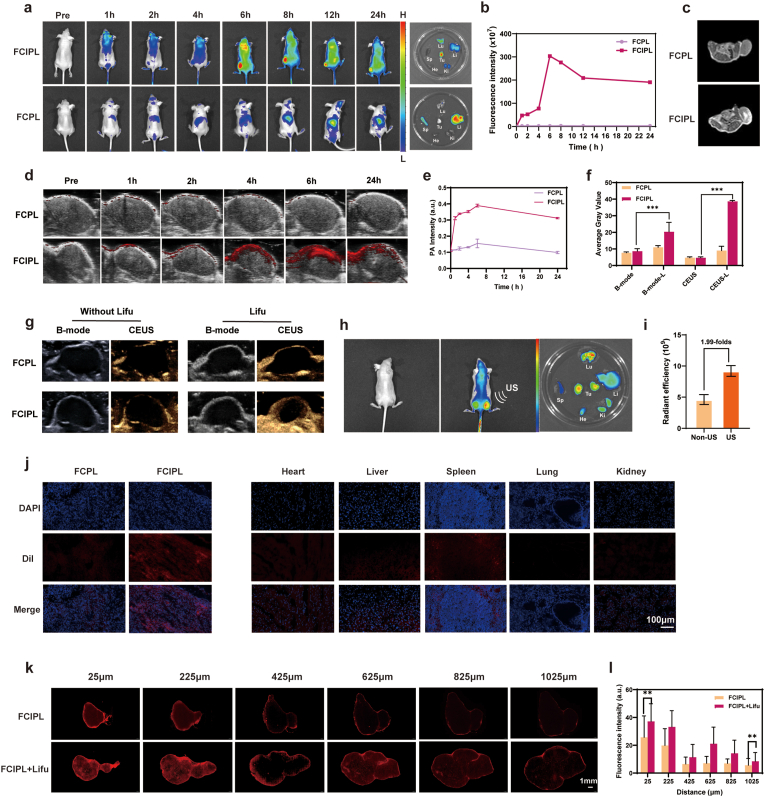
In vivo targeting, multimodal imaging and permeability of FCIPL NPs. a,b) In vivo fluorescence imaging and quantitative analysis of fluorescence intensity. c) In vivo MRI. d) In vivo photoacoustic imaging. e) PA signals of FCPL and FCIPL at rat tumor sites at different time points (n = 3). f) Relative gray values of B-Mode and CEUS images before and after Lifu irradiation of FCPL and FCIPL (n = 3). g) In vivo ultrasound imaging. h) In vivo fluorescence imaging and biological distribution of FCIPL in bilateral tumor-bearing rats on the right side (with US irradiation) and left side (without US irradiation. i) Quantitative analysis of fluorescence values of bilateral tumors (n = 3). j) Biodistribution of NPs in vivo. k,l) Penetration depth of FCIPL nanoparticles with or without ultrasonic irradiation in vivo (scale 1000 μm) and quantitative analysis of fluorescence intensity.

NPs with in vivo imaging capabilities can greatly improve the early diagnosis rates of diseases; therefore, we verified the multimodal imaging functions of FCIPL. As shown in Fig. 7a,b, after the FCIPL NPs were injected into nude mice, fluorescence signals began to appear in the tumor region at 2 h and then peaked at 6 h, after which they attenuated significantly at 24 h. However, there were no obvious signals in the tumor region of the FCPL group, which confirmed the tumor-targeting property of IR780. The optimal targeting time of FCIPL was 6 h, which provided a reference basis for in vivo treatment. The dissection of the nude mice revealed that the FCIPL fluorescence was located mainly in the lungs and tumors, indicating that the NPs were metabolized mainly through respiration. In addition, to confirm the targeting ability of FCIPL in vivo, we stained sections of the tumors and major organs taken 6 h after nanomaterial injection. As shown in Fig. 7j, a large amount of FCIPL was concentrated around the tumor cells, which strongly confirmed the targeting ability of FCIPL to achieve better therapeutic effects.

In vivo photoacoustic imaging revealed the same trend as in vivo fluorescence, with the photoacoustic signal peaking at 6 h and attenuating at 24 h. Moreover, the photoacoustic signal in the FCIPL group was much stronger than that in the FCPL group (Fig. 7d and e). According to fluorescence and photoacoustic imaging, the optimal targeting time after NPs injection was 6 h. Thus, we performed ultrasound imaging after irradiating the tumor with Lifu (3 W, 3 min) 6 h after material injection. As shown in Fig. 7f and g, there were no ultrasound signals in either 2D mode or CEUS mode before Lifu irradiation, but ultrasound signals appeared in the interior of the tumor on both sides after stimulation with Lifu in the FCIPL group. However, there was no difference before and after Lifu in the FCPL group, indicating that after the NPs encapsulating IR780 were excited by Lifu in the tumor region, PFP underwent ADV to produce microbubbles, which generated ultrasound signals due to the cavitation effect. We also performed MRI, and as shown in Fig. 7c, the T1-weighted image signal of the FCIPL group was greater than that of the FCPL group, which again confirmed the targeting ability of the FCIPL NPs.

### 2.10 Deep penetration of the FCIPL NPs in vivo

Encouraged by these successful in vitro experiments, we conducted in vivo experiments to investigate the penetration of FCIPL NPs into tumors. As shown in Fig. 7h and i, the fluorescence intensity of the ultrasound-irradiated tumors was 1.99 times greater than that of the nonirradiated tumors. The FCIPL NPs penetrated the tumor tissues from the top 25 μm to the center 1225 μm and were uniformly distributed in the center of the tumors upon ultrasound irradiation, whereas without ultrasound, the FCIPL NPs were distributed mainly on the surface of the tumors (Fig. 7k and l). The above experiments demonstrated that Lifu significantly enhanced the deep penetration of the FCIPL NPs in both 3D tumor spheres and solid tumors, effectively increasing the accumulation of the NPs in the tumor cells.

### 2.11 In vivo synergistic tumor treatment

**Fig. 8:**
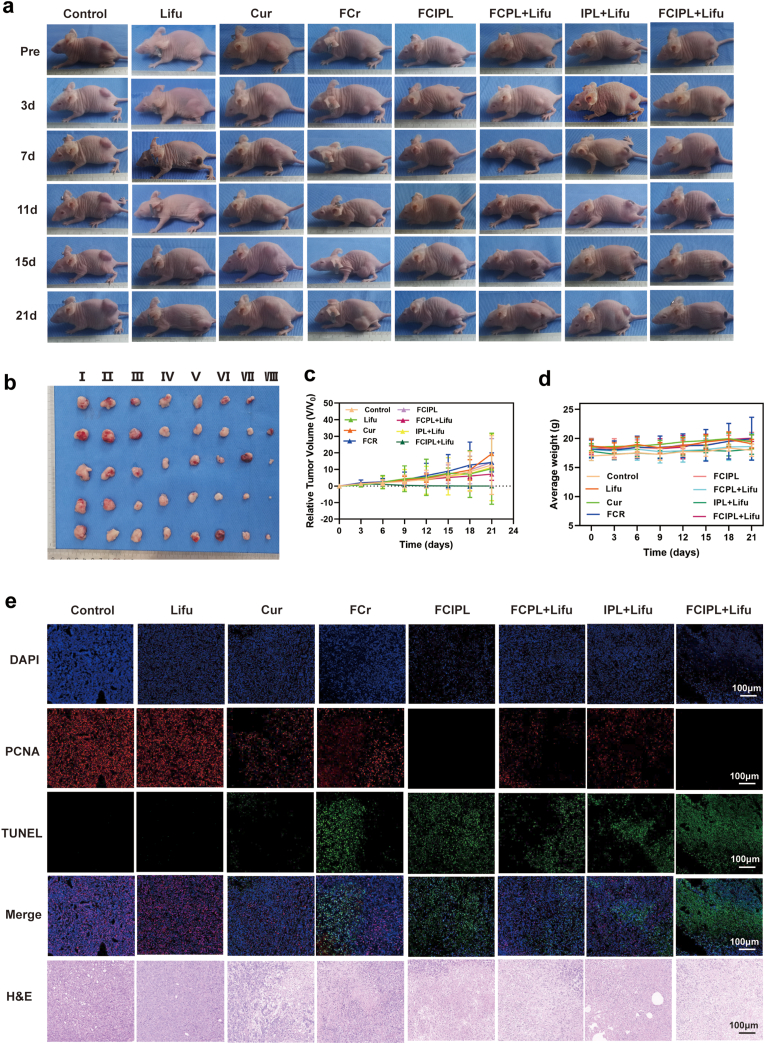
In vivo tumor therapeutic effects of FCIPL. a, b) Photographs of the different treatment groups before treatment, 3–21 days after treatment and the last image of the isolated tumor after 21 days of treatment, Ⅰ: Control, Ⅱ:Lifu, Ⅲ:Cur, Ⅳ:FCr, Ⅴ:FCIPL, Ⅵ:FCPL + Lifu, Ⅶ:IPL + Lifu, Ⅷ:FCIPL + Lifu. c) Changes in tumor size during the treatment with different treatments. d) Changes in body weight during treatment of tumor-bearing nude mice. e) PCNA, TUNEL and H&E staining of tumor tissue after different treatments. From top to bottom: DAPI-labeled nuclei (blue), PCNA-positive cells (red), TUNEL-positive cells (green), overlapping images and H&E staining, scale bar 100 μm.

Owing to the good in vivo targeting ability of FCIPL, we monitored the antitumor effect of synergistic treatment in BHT-101 hormonal model mice. Because in vivo fluorescence imaging revealed that the fluorescence intensity of the tumor peaked at 6 h after NPs injection, we irradiated the tumors with Lifu (3 W, 3 min) at this time point. After different treatments were given, we took photos of all the mice every 3 days over 21 days to observe tumor growth. At the end of the experiment, photos of the nude mice and their tumors at different stages of treatment were compared (Fig. 8a and b). Notably, the subcutaneously transplanted tumors in the FCIPL + Lifu group gradually shrank and were completely eradicated. The tumors on the back began to decrease in size on the 7th day of treatment, at which time the skin crusted, and the tumors completely disappeared on the 21st day. Observation continued until day 30 without any obvious recurrence, which confirmed that the combination of FCIPL and Lifu had a better therapeutic effect on ATC-transplanted tumors in nude mice. Certain therapeutic effects were also observed in the FCPL + Lifu and IPL + Lifu groups. The tumors in the Cur, FCr and FCIPL groups grew faster in the later stages of treatment, and there was no significant difference between the Lifu and control groups.

The therapeutic effect was evaluated by measuring the body weight and tumor volume of the nude mice in each group at different stages. There were no significant changes in the body weights of the nude mice throughout the entire treatment process (Fig. 8d). We then used the ratio of the front and rear tumor volumes (V/V_0_) as an indicator to evaluate the effect of tumor treatment. As shown in Fig. 8c, the FCIPL + Lifu group presented the strongest inhibitory effect. When the tumor volume is reduced to 0, the treatment effect is excellent. The inhibitory effects of the remaining groups were ranked from strong to weak as follows: FCPL + Lifu > IPL + Lifu > FCIPL > FCr > Cur > Lifu > control, which followed the same trend as the changes in the isolated tumors.

**Fig. 9:**
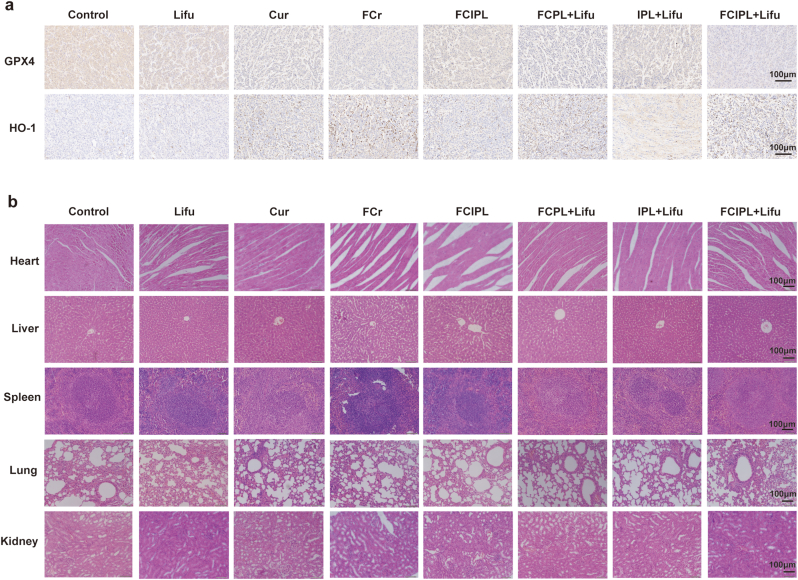
Tumor immunohistochemistry results and changes in major organs after treatment with different methods. a) Immunohistochemical examination of tumors after treatment with different methods, scale bar 100 μm. b) H&E staining of major organs after treatment with different methods; the scale bar is100 μm.

Tumor histological examination and immunohistochemical analysis (Fig. 8e) revealed that apoptotic cells (green fluorescence) were the most abundant in the FCIPL + LIFU group, and there were almost no proliferating cells (red fluorescence), demonstrating that this treatment had a good inhibitory effect on tumor growth. Additionally, the number of apoptotic cells in the remaining groups essentially corresponded to the changes in the tumors. The same trend was observed in H&E-stained sections. The cellular structures in the FCIPL + Lifu group were damaged, and coagulative necrosis occurred. The immunohistochemistry results (Fig. 9a) revealed a significant decrease in GPX4 expression and an increase in HO-1 expression in the FCIPL + Lifu group compared with the control group, which was consistent with the in vitro experimental results. The H&E-stained sections of the major organs in each group after treatment (Fig. 9b) revealed no obvious inflammatory changes in the nude mice in any of the groups, again demonstrating the in vivo safety of the FCIPL NPs in combination with SDT.

In this study, we prepared FCIPL nanoparticles with good safety and in vivo therapeutic effects. However, for subsequent clinical transformation, we still face challenges such as large-scale production, quality control and regulatory science, which require further detailed research. In the future, we may continue to conduct in-depth research on this nanomaterial and explore its combined efficacy with in treatments such as immunotherapy, gene editing therapy, and protein replacement therapy.

## 3 Conclusion

In summary, we successfully synthesized the cascade-penetrating metal–polyphenol ultrasonic molecular probe FCIPL. For diagnosis, the nanoprobe integrates multimodal (ultrasound, photoacoustic, fluorescence and MR) imaging. As a therapeutic, the probe can penetrate deep into tumor tissue step by step via specific targeting combined with UTMD technology. The drug was successfully delivered into the mitochondria of tumor cells under the cascade response of ultrasound in the tumor microenvironment, inducing the ferroptosis domino cascade, disassembling to release the Fenton reaction catalyst Fe^2+^, which combined with Cur to form a ferroptosis amplifier to induce an increase in LPOs, triggering ferroptosis, enhancing the effectiveness of SDT, and producing a waterfall-like therapeutic effect. The good therapeutic efficacy of FCIPL was validated in vitro and in vivo, which is expected to provide new ideas for the combined diagnosis and treatment of ATC.

## 4 Materials and methods

### Materials

Dipalmitoyl phosphatidylcholine (DPPC), cholesterol (CHO), 1,2-distearoyl-sn-glycero-3-phosphoethanolamine-N-[methoxy(polyethylene glycol)-5000] (DSPE-PEG-5000) and IR780 iodide (IR780) were purchased from Xi'an Ruixi Biological Technology Co., Ltd. (Xi'an, China). Perfluorohexane (PFP) was acquired from Macklin (Shanghai, China). Curcumin, 1,3-Diphenylisobenzofuran (DPBF), and dichlorofluorescin diacetate (DCFH-DA) were obtained from Sigma‒Aldrich. Iron chloride hexahydrate (FeCl_3_·6H_2_O) and Singlet Oxygen Sensor Green (SOSG) were obtained from Thermo Fisher (USA). Polyvinyl pyrrolidone (PVP), methylene blue (MB), GSH, 5,5′-dithiobis (2-nitrobenzoic acid) (DTNB), Vitamin C (VC) and vitamin E (VE) were obtained from Aladdin Industrial Corporation (Shanghai, China). Cell-counting Kit-8 (CCK-8), Ferrostatin-1 (Fer-1), CAM, PI, FerroOrange, and Liperfluo were purchased from Dojindo Molecular Technologies, Inc. (Japan), and 1,1′-dioctadecyl-3,3,3′,3′-tetramethylindotricarbocyanine iodide (DiR), 1,1′-dioctadecyl-3,3,3′,3′, tetramethyl indocarbocyanine perchlorate (DiI), 4′,6-diamidino-2-phenylindole (DAPI), MitoTracker green, and the BCA protein assay kit were obtained from Beyotime (Shanghai, China). A reduced GSH assay kit was purchased from Nanjing Jiancheng Bioengineering Institute. JC-1, AC-DEVD-CHO (APO), necrostatin-1 (Nec-1), 3-methyladenine (3 MA), and deferoxamine (DFO) were obtained from Selleck Chemicals (Houston, USA). DMEM, fetal bovine serum (FBS) and PBS were purchased from Gibco. Heme oxygenase 1 antibody (HO-1), glutathione peroxidase 4 antibody (GPX4), Anti-xCT/SLC7A11 antibody and Anti-FACL4 antibody were obtained from Abmart (China). PCNA and TUNEL detection kits were procured from Servicebio Technology Co. Ltd. (Wuhan, China).

### Methods

#### Synthesis of FCr-PFP@IR780-LIP

PVP (66 mg) was dissolved in 5 mL of methanol, to which 20 mg of FeCl_3_ in 1 mL of methanol was added dropwise with stirring. Subsequently, 10 mg of Cur was mixed with 1 mL of methanol, and this solution was then added dropwise to the above mixture with magnetic stirring for 3 h. The resulting FCr solution was dialyzed against double distilled water overnight to remove the methanol.

The NPs were subsequently prepared via a vacuum film-sonic shock method. First, 10.0 mg of DPPC, 5.0 mg of CHO, 5.0 mg of DSPE-PEG-5000, and 0.5 mg of IR780 were dissolved in 10.0 mL of CHCl_3_ in a 100 mL flask. The organic solvent was removed by rotary evaporation at 45 °C for 20 min. Subsequently, the prepared film was hydrated with 4 mL of PBS (pH = 7.4). Afterward, 100 μL of PFP and 50 μL of FCr were added dropwise to the above suspension under sonication for 5 min (pulse duration, 5 s; pulse interval, 5 s) in an ice bath. The resulting NPs were stored at 4 °C. Additionally, the FCPL and IPL preparation methods were similar to the aforementioned process except that IR780 and FCr were not added. DiI/DiR-labeled NPs were fabricated via the same method in which 5 μL of DiI/DiR was added to CHCl_3_.

### Characterization

TEM (JEM-2100F) was used to analyze the morphology, size, and distribution of FCIPL and FCr as well as the ADV of FCIPL following Lifu irradiation. Additionally, an optical microscope (CKX41; Olympus, Tokyo, Japan) was used to observe the morphology and distribution of FCIPL. The hydrated particle size, polydispersity index (PDI), and zeta potential were analyzed with a Malvern particle size analyzer (DLS; Nano, ZS90, Malvern Instruments, UK). The stability of FCIPL was assessed by measuring the average hydrated particle size over a 7-day period at 4 °C. The chemical groups and bonds within the material were identified via Fourier transform infrared (FTIR) spectroscopy (Bruker VERTEX 80v), and XPS (Thermo VG Scientific ESCALAB 250) was used to analyze the elemental composition and valence state. The absorption spectra of the samples were acquired via UV spectrophotometry (UV2550, Shimadzu, Japan). To calculate the encapsulation efficiency (EE) and loading capability (LC) of IR780 and FCr, the absorption spectra of samples encapsulating IR780 and FCr were analyzed via standard curves, which were generated from the absorption spectra of IR780 and FCr at various concentrations. The following equations were used for analysis:

EE= (entrapped IR780 (or FCr)/total IR780 (or FCr) input) × 100 %; and

LC= (entrapped IR780 (or FCr)/total weight of the NPs) × 100 %.

### Consumption of GSH and release of Fe^2+^

After ultrasonic irradiation of the dialysis bag containing 2 mL of FCIPL solution (power, 3 W; duration, 2 min), 58 mL of GSH release medium (10 mM, containing 1 % DMSO) was added, the mixture was shaken at 400 rpm, 3 mL of the solution outside the dialysis bag was removed at different time points, and the medium was replenished. A 1 mg/mL o-phenanthroline aqueous solution was added to the extracted solution, and a UV spectrophotometer was used to detect the absorbance of the solution at 510 nm and we included the Cur-PFP@IR780-LIP group as a negative control. Upon monitoring Fe^2+^ by ICP-AES at different time points. Moreover, 10 μM DTNB solution was added to the removed solution, and the intensity of the UV absorption peak at 412 nm was compared at different time points to verify the consumption of GSH.

### Detection of reactive oxygen species (ROS)

200 μL of FCIPL (5 mg/mL) and 3 mL of GSH solution (10 mM) were mixed and placed in a six-well plate. Under ultrasonic irradiation for 2 min in the dark (power 3 W) and incubation for 1 h, 20 μL of MB solution (1 mg/mL) and 5 μL of H_2_O_2_ solution (1 mM) were added, and the mixture was shaken for 2 h. A UV spectrophotometer was used to detect changes in the intensity of the absorption peak of MB.

Next, 10 μL of a solution of SOSG in methanol (50 μM) was added to 3 mL of FCIPL solutions of different concentrations, 10 mM GSH was added, and the mixture was irradiated with Lifu (power 3 W) for 2 min in the dark. A fluorescence spectrophotometer was used to detect the fluorescence intensity at 525 nm. Similarly, 40 μL of DPBF solution (8 mM) was added to 3 mL of FCIPL (1 mg/mL), and 10 mM GSH was also added. The mixture was irradiated with Lifu (power 3 W) for different durations, after which a UV spectrophotometer was used to measure the change in the intensity of the absorption peak at 398 nm. In accordance with the same method, TEMP (100 mM) was added to FCIPL solution (1 mg/mL), and the generation of singlet oxygen was detected by ESR after Lifu irradiation.

### Cell culture and construction of the BHT-101 tumor-bearing mouse model

The human ATC cell line BHT-101 and human vascular endothelial cells (HUVECs) were obtained from Wuhan Punosai Life Science and Technology Co., Ltd., China. The BHT-101 cells and HUVECs were cultured in DMEM with 10 % FBS, 100 U/mL penicillin and 100 mg/mL streptomycin in a humidified incubator containing 5 % CO_2_ and maintained at 37 °C.

The use of all the animals (4-week-old female Kunming mice and 4-week-old female BALB/c nude mice weighing 14–16 g) was approved by the ethical application of Laboratory Animal Center, College of Life Science, Jilin University, and the ethical approval number was 2022-XWPZSY1002. To establish the ATC subcutaneous tumor model, 100 μL of BHT-101 cells (5 × 10^7^/mL) were injected subcutaneously into the left posterior dorsal region of the nude mice, and in vivo experiments were performed when the tumor volume reached 100 mm^3^.

### In vitro multimodal imaging with FCIPL

**Ultrasound imaging:** The appropriate material (FCIPL NPs in the experimental group and PBS in the control group) was added to agarose gel model wells, and ultrasound imaging was carried out using an ultrasound diagnostic instrument (Aixplorer, Supersonic Imagine) and a line array probe (L15-4), including B-mode and CEUS mode. Ultrasound images before Lifu irradiation were captured as a control, and images were captured again after 3 min of Lifu irradiation at 3 W. In the other group, 2 mg/mL FCIPL NPs were added to the wells of the gel model and irradiated with different ultrasound intensities for 5 min. Ultrasound images were acquired at different time points, and the average gray value of the region of interest (ROI) was measured with DFY software for quantitative analysis.

**Photoacoustic imaging:** A 1 mg/mL FCIPL suspension was added to a thin tube and scanned using a photoacoustic imaging system (Vevo LAZR, Canada) in the full range of 700–950 nm to construct the corresponding photoacoustic signal curve.

The FCIPL solution was then diluted to different concentrations, and a photoacoustic instrument was set to apply a pulsed laser at a wavelength of 780 nm to obtain photoacoustic images at each concentration. The instrument was used to record the average relative photoacoustic signal intensity in the ROI, from which a standard curve was plotted.

**MRI:** One milliliter of each concentration of FCIPL solution was added to separate 1.25 mL EP tubes, with PBS used as a control, and T1-weighted images were obtained via MRI.

**Fluorescence imaging:** FCIPL NPs (2 mg/mL; experimental group) and PBS (control group) were added to 1.5 mL EP tubes, and a fluorescence imaging instrument (Fx7 IR Spectra, Vilber Lourmat, France) was used for image collection.

### In vitro uptake and targeting

BHT-101 cells were cultured in confocal dishes for 24 h at a density of 1 × 10^5^. DiI-labeled FCPL and FCIPL NPs were suspended in serum-free DMEM (at a concentration of 100 μg/mL) in the dark and added to the dishes for 0.5, 1, 2, or 3 h of incubation. Then, the cells were washed with PBS and fixed with paraformaldehyde, and the fixed cells were collected and stained with 100 μL of DAPI. Finally, the cells were observed via CLSM. Similarly, BHT101 cells were inoculated into six-well plates at a density of 2 × 10^5^, and 1 mL of DiI-labeled FCIPL NPs (100 μg/mL) was added to each well, followed by incubation for the same amount of time, digestion with EDTA-free trypsin, centrifugation, and resuspension in PBS. The cellular uptake of the NPs was subsequently analyzed via flow cytometry.

To further determine the subcellular targeting ability of the FCIPL NPs, 100 μL of DiI-labeled FCPL or FCIPL NPs (100 μg/mL) were added to confocal Petri dishes containing BHT-101 cells in the dark, and after coincubation for 3 h, the cells were stained with MitoTracker Green for 20 min and washed with PBS, after which the enrichment of the NPs in the mitochondria was observed via CLSM.

### Deep penetration of FCIPL in vitro

To evaluate the ultrasound penetration performance, we indirectly measured ROS production in a black agar gel model. 200 μL of FCIPL suspension was added to the model wells, followed by the addition of 1 μL of SOSG; an equal amount of PBS was added to the control group. Then, the wells were irradiated with Lifu for 2 min. Images were captured by an in vivo fluorescence imaging system, and the fluorescence intensities were measured and analyzed (ex = 488 nm, em = 525 nm).

To evaluate the penetration of FCIPL NPs in vitro, we constructed a 3D tumor sphere model for observation. The experiment was divided into three groups: FCPL, FCIPL and FCIPL + Lifu. BHT-101 cells (6 × 10^4^/well) were cultured in spherical microtiter plates for one week, washed with PBS and incubated with 100 μg/mL DiI-FCIPL or DiI-FCPL NP suspensions instead of medium for 2 h. The target group was irradiated with Lifu (2 W) for 3 min, incubated again for 1 h, fixed with paraformaldehyde, stained with DAPI for 20 min, and washed with PBS. Then, the tumor spheres were transferred to a confocal dish, and the penetration of FCIPL into the tumor spheres was observed via confocal laser scanning microscopy (CLSM). Images were acquired at 20 μm intervals from the apex to the maximal surface in an XYZ-3D-stack.

### In vitro cytotoxicity assay and mechanism of action

The cytotoxicity of FCIPL was examined via CCK-8 assays. HUVECs and BHT-101 cells were inoculated in 96-well plates at a density of 4 × 10^3^ cells per well, and after the cells were cultured for 24 h to allow wall attachment, different concentrations of FCIPL NPs were added to both types of cells. The survival rates of the cells were analyzed via CCK-8 assays after 24 h of incubation. To determine the optimal Lifu parameters for tumor cell killing, we added 0.5 mg/mL FCIPL NPs to 96-well plates containing BHT-101 cells for 2 h of incubation followed by Lifu irradiation at different powers (1, 2, or 3 W) for 30, 60, 90, 120, or 180 s. After 24 h of incubation, the survival rates of the cells were determined. We also investigated the toxicities of different treatments on BHT-101 cells in the following 8 groups: the control, Lifu, FeCl_3_, Cur, FCr, IPL, FCIPL, FCPL + Lifu, IPL + Lifu and FCIPL + Lifu groups. Equivalent concentrations of FCr and IR780 were maintained in each group, and the FCIPL concentration was 1 mg/mL. Cell survival was analyzed via CCK-8 assays after treatment. Lifu was performed at 3 W for 3 min. Similarly, BHT-101 cells were inoculated in confocal dishes at a density of 1 × 10^5^ and divided into the same eight groups. A mixture of the dyes CAM and PI was then added proportionally, and the distributions of live and dead cells were determined via CLSM after 30 min of staining.

To investigate the mechanism of FCIPL cytotoxicity, we added Fe-1 (1 μM), VE (200 μM), VC (50 μM), DFO (5 μM), ADC (50 μM), Nec-1 (1 μM), or 3 MA (10 μM) to FCIPL + Lifu-treated BHT-101 cells in 96-well plates and then measured the cellular activity with CCK-8 assays to analyze the mechanism of action of the NPs after 24 h of incubation.

### Determination of intracellular ROS levels

To evaluate intracellular ROS production after different treatments, we inoculated BHT-101 cells in confocal dishes at a density of 1 × 10^5^ and treated the cells with DMEM, Lifu, Cur, FCr, FCIPL or FCIPL + Lifu (at an equivalent dose of 200 μg/mL FCIPL; the Lifu parameters were the same as above). After 24 h of treatment, the cells were stained with the ROS detection probe DCFH-DA for 20 min and washed three times with PBS, and the intracellular ROS content was determined via CLSM. In addition, flow cytometry was used to further analyze the ROS levels in BHT-101 cells after NPs treatment. BHT-101 cells were inoculated in six-well cell culture plates and incubated for 24 h. The cells were treated as above for 24 h, digested with trypsin, centrifuged, resuspended, and then stained for 20 min. Flow cytometry was used to quantify the intracellular ROS levels.

### Determination of the intracellular Fe^2+^ level

To detect the intracellular Fe^2+^ level, we inoculated BHT-101 cells in confocal dishes for 24 h of incubation after different treatments (DMEM, Lifu, Cur, FCr, FCIPL and FCIPL + Lifu) with the same dose of 200 μg/mL FCIPL and the same Lifu parameters as above. Then, the cells were washed with PBS, FerroOrange stain was added for 30 min of incubation, and finally, the intracellular Fe^2+^ content was determined via CLSM.

### Mitochondrial membrane potential assay

To detect changes in the intracellular mitochondrial membrane potential, we inoculated BHT-101 cells in confocal dishes for 24 h of incubation. After treatment (DMEM, Lifu, Cur, FCr, FCIPL, and FCIPL + Lifu), the cells were incubated for 24 h, washed with PBS and stained with JC-1 working solution for 20 min. Intracellular fluorescence was observed via CLSM.

### Intracellular LPO accumulation assay

BHT-101 cells were inoculated in six 20 mm confocal dishes, incubated for 24 h, subjected to different treatments (DMEM, Lifu, Cur, FCr, FCIPL and FCIPL + Lifu), washed with PBS after 24 h of incubation, and stained with Liperfluo reagent for 20 min, after which the intracellular LPO content was determined via CLSM.

### Determination of the intracellular protein expression levels

BHT-101 cells were inoculated into six-well cell culture plates and incubated for 24 h. The cells were collected after treatment in the same groups described above. The cells were lysed in RIPA lysis buffer containing protease and phosphatase inhibitors to collect the proteins, and the protein content of each group was determined with a BCA protein quantification kit. The proteins were then electrophoretically separated, and transferred to a PVDF membrane, and blocked with milk for 1 h. After washing three times, the primary antibodies (GPX4, HO-1, ACSL4, SLC7A11 and β-actin) were added for incubation overnight at 4 °C, followed by coincubation with a fluorescent secondary antibody in the dark for 1 h. After an additional washing step, visualization and quantitative analysis were performed with an Odyssey Dual-Color Infrared Laser Imaging System.

### Changes in mitochondrial morphology

BHT-101 cells were inoculated in six-well cell culture plates and treated with PBS or FCIPL + Lifu. The NPs concentration and Lifu parameters were the same as above, and the cells were collected after 24 h of incubation. After rinsing the cells 3 times with PBS, 2.5 % glutaraldehyde fixative was added, and the cells were incubated at 4 °C overnight. The cells were then washed 3 times with 0.1 M phosphoric acid rinse solution, fixed with 1 % osmium acid for 2 h, dehydrated with gradient ethanol and embedded. Ultrathin sections were obtained with an ultrathin sectioning machine (Leica UC7, Leica) and then double-stained with 3 % uranyl acetate-lead citrate. The morphology of the subcellular structures was subsequently observed via TEM.

### mRNA sequencing and analysis

BHT-101 cells were inoculated into six-well plates and treated with PBS or FCIPL + Lifu with the same parameters as before. After 24 h of culture, RNA was extracted from the samples with TRIzol, and the genomic DNA was removed using DNaseI. An RNA-seq transcriptome library was established, and high-throughput sequencing was subsequently performed.

### In vivo biosafety analysis of FCIPL

Healthy female Kunming mice aged 4 weeks were randomly divided into 6 groups of 5 rats each, with 1 group being the control group and the remaining 5 groups being the experimental groups. In each experimental group, 200 μL of FCIPL (5 mg/mL) was injected via the tail vein, and orbital blood was collected on days 1, 3, 7, 15, and 30 after injection for routine blood tests, including RBCs, WBCs, Lymphs, PLTs, HGB, HCT, MCV, and MCHC. The blood samples were centrifuged, and the supernatants were separated for liver function (AST and ALT), kidney function (CREA, UREA), and cardiac enzyme (CK) analyses. Finally, the mice were sacrificed, and 3 mice from each group were randomly dissected. The major organs, such as the heart, liver, spleen, lung and kidney, were collected, embedded in paraffin wax and stained with H&E, and the samples were observed with a microscope.

### In vivo imaging experiments

**Fluorescence imaging:** Tumor-bearing nude mice were randomly divided into two groups and injected with 200 μL of DiR-labeled FCPL or FCIPL (5 mg/mL) NPs suspension via the tail vein. Fluorescence images were collected with a small animal in vivo fluorescence imager at 0, 1, 2, 4, 6 and 24 h after injection. After 24 h of imaging, the nude mice were dissected, and the tumors, heart, liver, spleen, lungs, and kidneys were removed for fluorescence imaging.

**Photoacoustic imaging:** The experimental method was the same as that for fluorescence imaging, and a photoacoustic imager was used to collect and analyze the images.

**Ultrasound imaging:** FCPL or FCIPL NPs (200 μL) were injected via the tail vein, and the optimal therapeutic time was selected according to the results of the photoacoustic imaging experiments. Ultrasound images before Lifu irradiation of the tumor site were collected as a control group, and then the tumor sites of the mice were irradiated with 3 W of Lifu for 3 min 2D ultrasound and CEUS imaging were performed on the tumors.

**MRI**: First, T1-weighted MRI was performed on tumor-bearing mice before NPs injection as a control, and then, 200 μL of FCIPL NPs was injected via the tail vein. Imaging was then performed again 6 h after injection to compare the differences between the before and after images.

### In vivo targeting experiments

In vivo targeting of the FCIPL NPs was observed by in vivo fluorescence imaging. The experimental method was the same as that described above. To further analyze the in vivo targeting ability of FCIPL, DiI-labeled FCIPL NPs were injected into the mice via the tail vein, and the mice were sacrificed 6 h later. The tumors and organs of the mice were removed and dissected. The isolated organs and tumors were embedded in paraffin, sectioned and stained with DAPI, and CLSM was used to observe the in vivo distribution of the FCIPL NPs in each section.

### In vivo experiments on the penetration of FCIPL NPs in deep tumors

Bilateral tumor-bearing nude mice were injected with 200 μL of DiI-FCIPL NPs via the tail vein, and after 6 h, one side of the tumor was irradiated with 2 W of Lifu for 3 min, and the other side was left untreated. The mice were placed in a fluorescence imaging instrument for imaging, and the difference in fluorescence intensities between the two sides of the tumors was observed to evaluate the effect of ultrasound on NPs penetration into the deeper parts of the tumors. The mice were subsequently sacrificed, and the bilateral tumors were dissected, embedded in paraffin, sectioned in layers, and preserved at 300 μm intervals. The degree of FCIPL penetration into the tumor tissue was subsequently assessed via CLSM.

### In vivo synergistic tumor treatment

BHT-101 tumor-bearing nude mice with a tumor volume of approximately 100 mm^3^ were randomly divided into 8 groups of 5 mice each as follows: (1) control, (2) Lifu, (3) Cur, (4) FCr, (5) FCIP, (6) FCPL + Lifu, (7) IPL + Lifu, and (8) FCIPL + Lifu. The corresponding materials were injected into the tumor-bearing mice via the tail vein at an FCIPL concentration of 5 mg/mL. Six hours after injection, the nude mice were anesthetized via an intraperitoneal injection of 100 μL of 1 % sodium pentobarbital. After anesthesia was complete, groups (2), (6), (7), and (8) were treated with Lifu (3 W, 3 min). The distance between the probe and the tumor was adjusted to ensure that the focus of the probe was located at the tumor site. Treatment was performed every 3 days on days 1, 4, 7, 10, 13, 16, 19 and 22. The health status of the nude mice was observed and recorded, the tumor sites of the nude mice were photographed, the tumor parameters and body weights of all nude mice were measured, and the tumor volume (V) was calculated using the following formula: V (mm^3^) = length × width [2]/2. The change in tumor volume was calculated via comparison with the initial tumor volume (V_0_) before treatment (V/V_0_). On the 22nd day, the nude mice were sacrificed and dissected, and the tumors were removed, photographed and weighed. H&E, PCNA and TUNEL staining were performed to observe the apoptosis and proliferation of tumor cells in each group, and then immunohistochemistry was performed using GPX4 and HO-1 antibodies to analyze the expression of the corresponding antigens in the tumors. Moreover, major organs, such as the heart, liver, spleen, lungs and kidneys, were removed and stained with H&E to observe histological changes.

### Statistical analysis

The experimental data in this study were statistically analyzed with SPSS 22.0 software and are presented as the means±standard deviations (SDs). Repeated measures were compared via independent samples t tests and one-way ANOVA, and differences were considered statistically significant at P < 0.05 (∗P < 0.05, ∗∗P < 0.01, ∗∗∗P < 0.001).

## CRediT authorship contribution statement

**Peng Dong:** Writing – original draft, Resources, Methodology, Investigation, Formal analysis, Data curation. **Yun-Bo Chi:** Validation, Software, Methodology. **Deng-Ke Teng:** Validation, Supervision, Methodology. **Yuan-Qiang Lin:** Software, Methodology. **Ling-Yu Zhu:** Supervision, Software, Methodology. **He-Qun Li:** Methodology, Investigation. **Jia-Yu Yang:** Software, Methodology. **Jia-Rui Du:** Methodology. **Zong-Tao Zhang:** Methodology. **Hai-Tao Ran:** Investigation. **Guo-Qing Sui:** Writing – review & editing, Validation, Supervision, Resources. **Hui Wang:** Writing – review & editing, Visualization, Methodology, Investigation, Funding acquisition. **Qi-Meihui Wang:** Writing – review & editing, Validation, Supervision, Investigation.

## Ethical approval

This study was approved by the ethical application of the Laboratory Animal Center, College of Life Science, Jilin University, and the ethical approval number is 2022-XWPZSY1002.

## Declaration of competing interest

The authors declare that they have no known competing financial interests or personal relationships that could have appeared to influence the work reported in this paper.

## Data Availability

Research data are stored in an institutional repository and will be shared upon request to the corresponding author.
