# Supplementary material for: Cascade-penetrating domino-ferroptosis nano inducer synergizes with sonodynamic therapy for anaplastic thyroid cancer
Source: Mater Today Bio. 2025 Aug 16;34:102206. doi: 10.1016/j.mtbio.2025.102206 (PMC12395522; doi:10.1016/j.mtbio.2025.102206)
Supplement: Supplementary file 1 [file mmc1.docx]

Supporting Information

**Cascade-Penetrating Domino-Ferroptosis Nano Inducer Synergizes Sonodynamic Therapy For Anaplastic Thyroid Cancer**

Peng Dong^1^, Yun-Bo Chi^2^, Deng-Ke Teng^1^, Yuan-Qiang Lin^1^, Ling-Yu Zhu^1^, He-Qun Li^1^, Jia-Yu Yang^1^, Jia-Rui Du^1^, Zong-tao Zhang^3^, Haitao Ran^4^, Guo-Qing Sui^*^, Hui Wang^1*^, Qi-Meihui Wang^1*^

**Affiliation:**

1. Department of Ultrasound, China-Japan Union Hospital of Jilin University, Changchun, Jilin, 130033, China.
2. Department of Radiation Oncology, China-Japan Union Hospital of Jilin University, Changchun, Jilin, 130033, China.
3. State Key Laboratory of Inorganic Synthesis and Preparative Chemistry, College of Chemistry, Jilin University, Changchun, Jilin, 130012, China.
4. Institute of Ultrasound Imaging, The Second Affiliated Hospital of Chongqing Medical University, Chongqing 400010, China.

**Keywords: Cascade, Penetrating, Ferroptosis, Sonodynamic therapy, Anaplastic thyroid cancer.**

**Supplementary Figures**


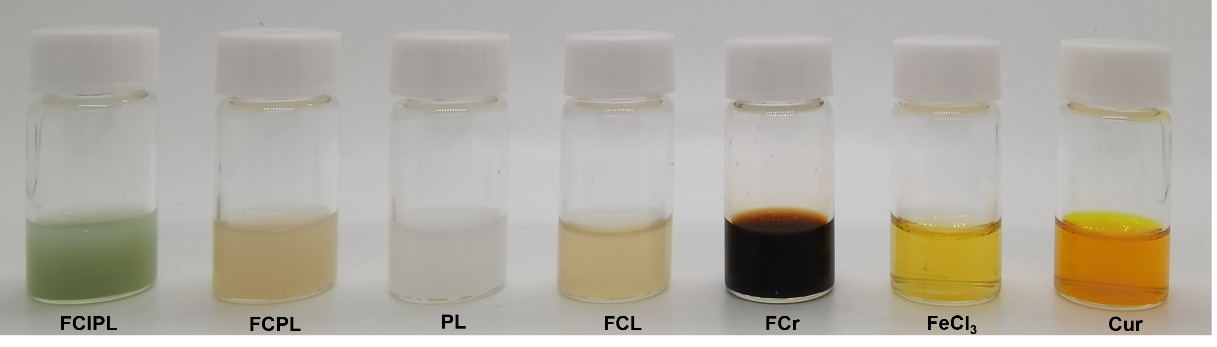

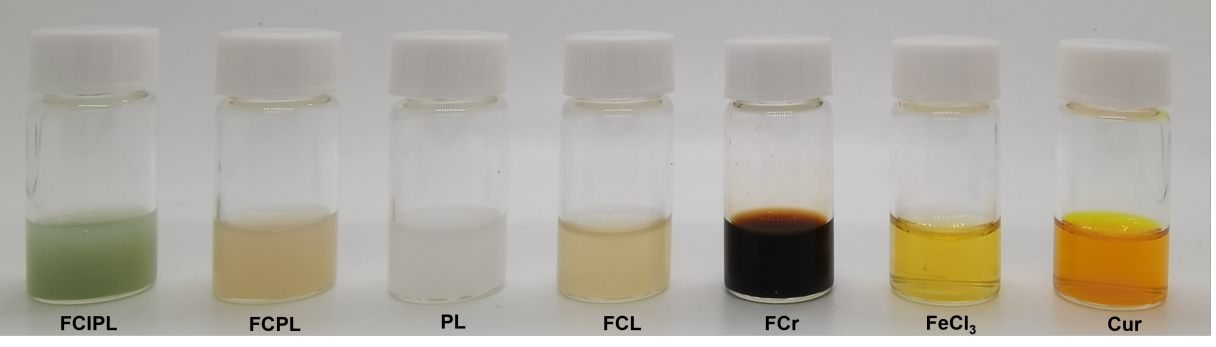


**Figure S1.** Pictures of the appearance of FCIPL, FCPL, FCr, FeCl_3_, and Cur solutions from left to right.


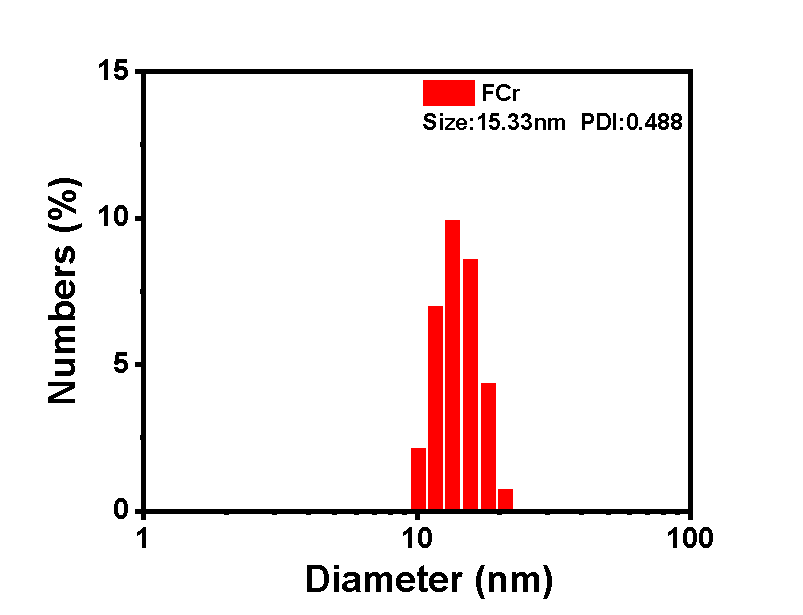


**Figure S2.** Particle size distribution of FCr.





**Figure S3.** Particle size distribution of FCPL.

| **Groups** | **Size (nm)** | **PDI** | **Zeta (mV)** |
| --- | --- | --- | --- |
| **FCr** | **15.33±2.336** | **0.488** | **26.6±14.1** |
| **FCPL** | **202.3±100.2** | **0.345** | **-11.5±5.41** |
| **FCIPL** | **218.6±55.77** | **0.04** | **-15.4±7.66** |

**Figure S4.** Particle size, PDI and potential characteristics of FCr, FCPL and FCIPL.





**Figure S5.** UV-visible absorption spectrum of Fe^2+^ released by Cur-PFP@IR780-LIP in GSH solution at different time.





**Figure S6.** DTNB detection of the UV-visible absorption spectrum of GSH consumption of FCIPL at different times.


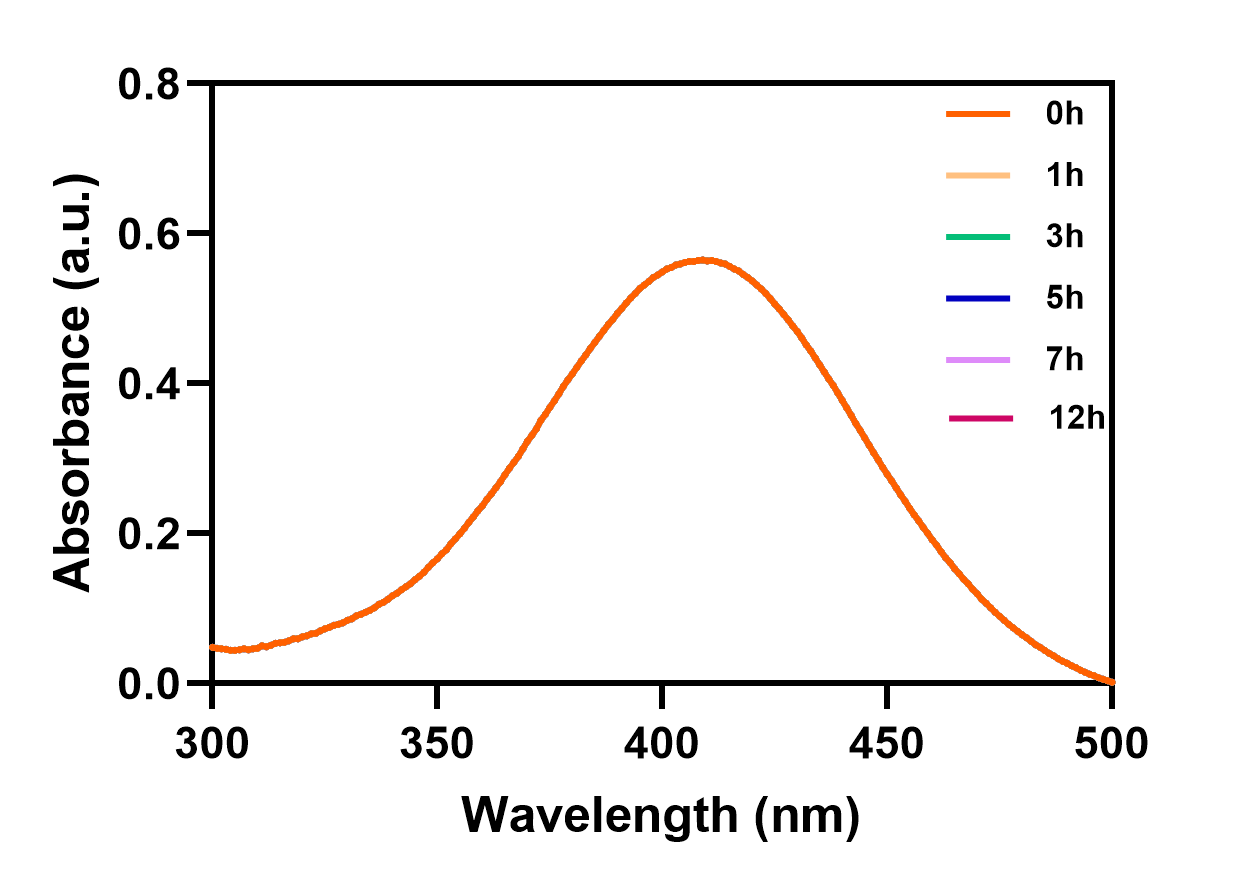


**Figure S7**.DTNB detection of the UV-visible absorption spectrum of GSH consumption of Cur-PFP@IR780-LIP at different times.


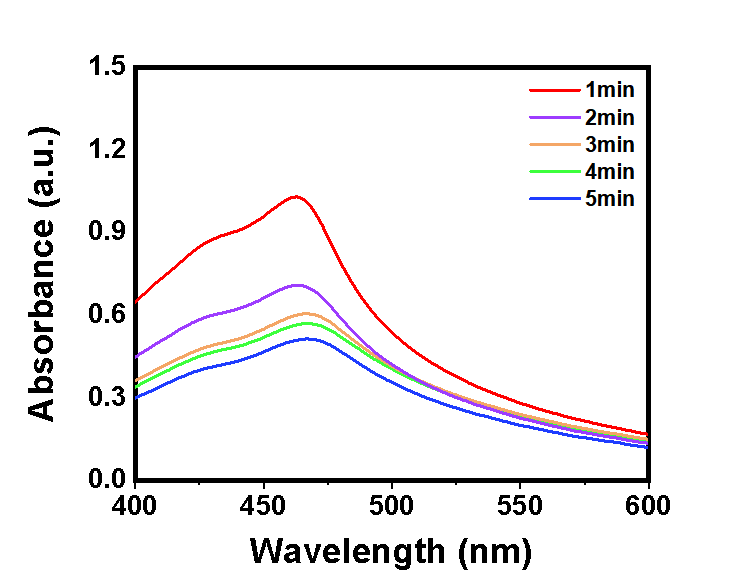


**Figure S8**. Detection of ^1^O_2_ produced by Lifu irradiation at different times using DPBF.


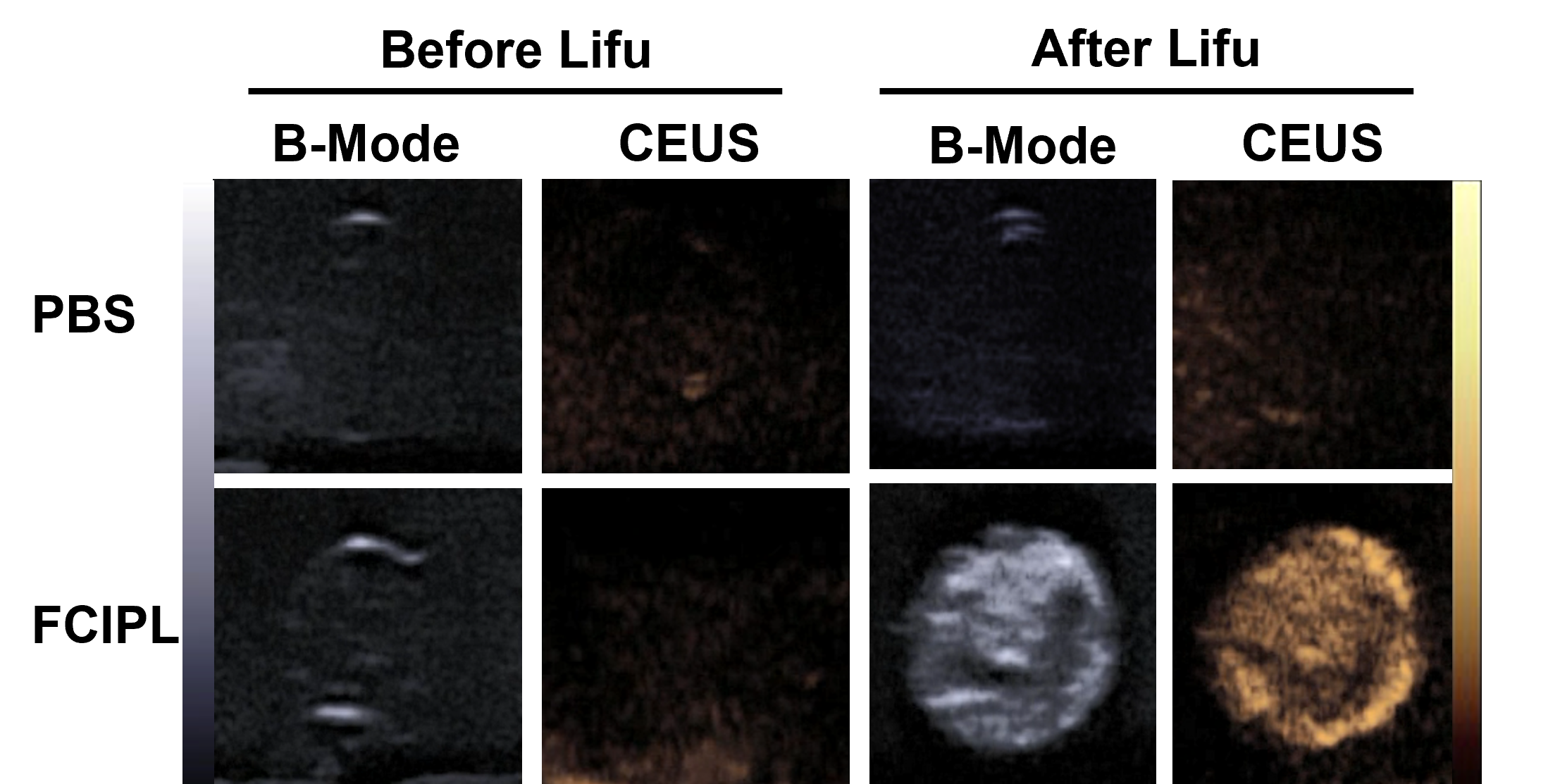


**Figure S9**. In vitro ultrasound imaging. Two-dimensional ultrasound images and contrast-enhanced ultrasound images before and after Lifu irradiation


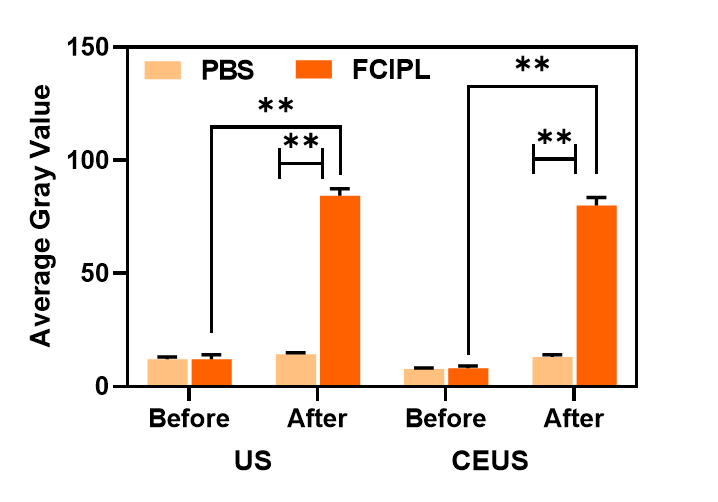


**Figure S10**. Gray values of ultrasound images in different groups (n=3, ***P < 0.01).


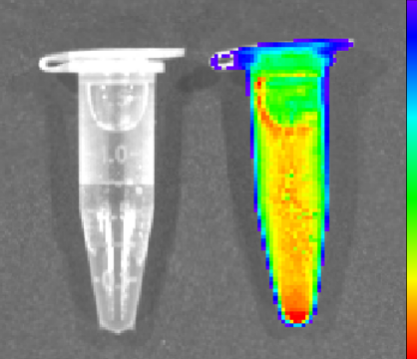


**Figure S11**. In vitro fluorescence imaging of different concentrations of FCIPL.


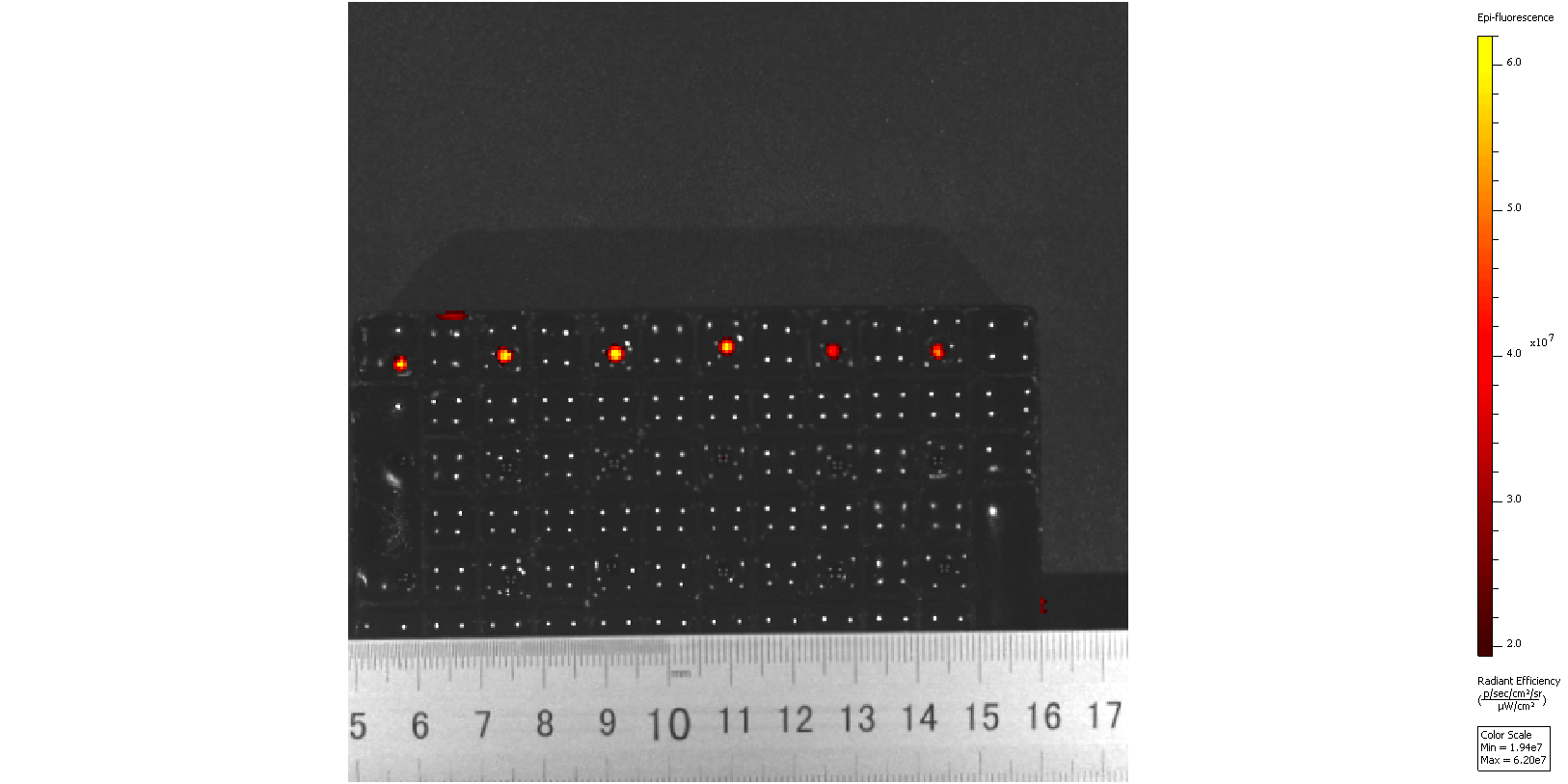

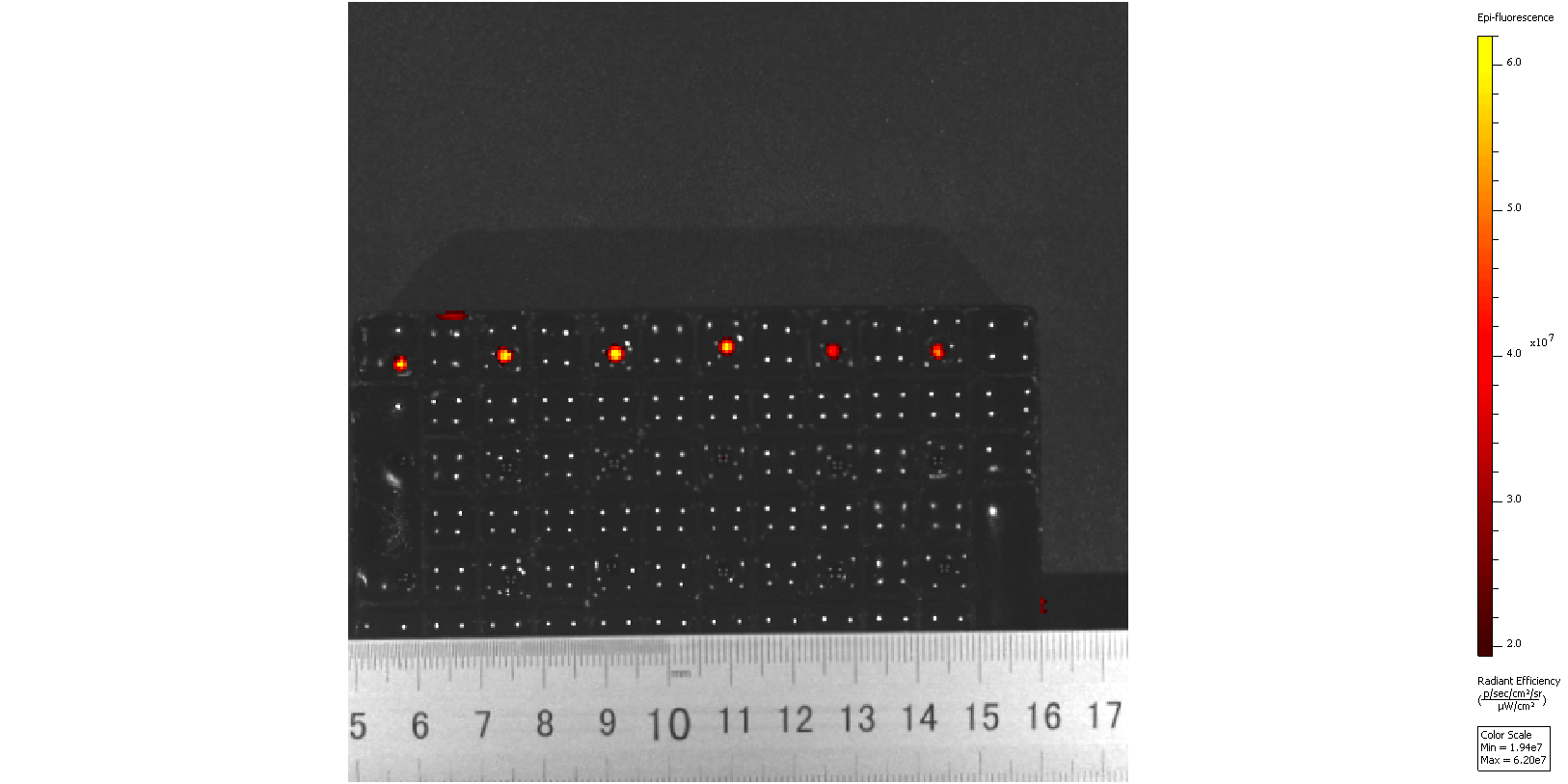


**FCIPL**

**PBS**

**Lifu**

**Figure S12**. In vitro permeation fluorescence image of FCIPL. Fluorescence images of FCIPL and PBS suspension containing SOSG after ultrasonic excitation;


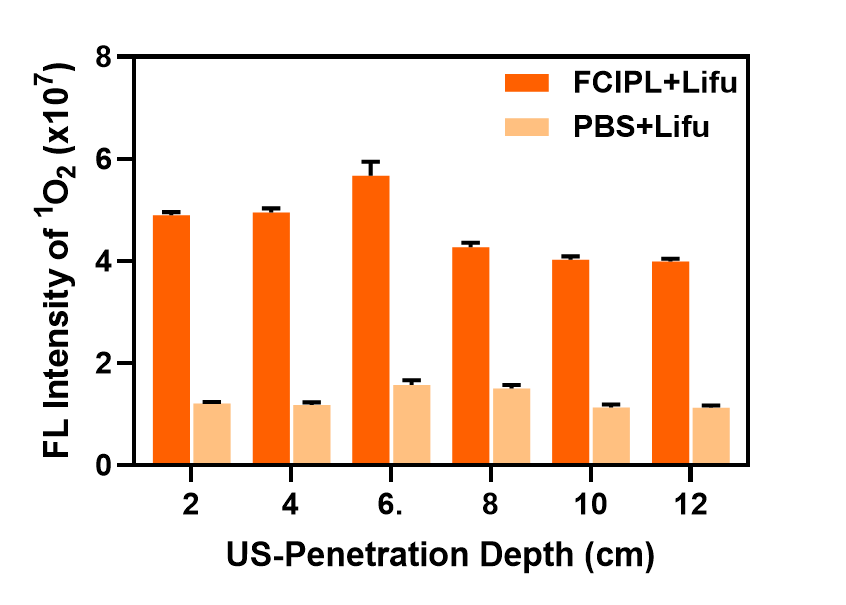


**Figure S13**. Quantitative analysis of fluorescence values at different depths after ultrasonic irradiation.


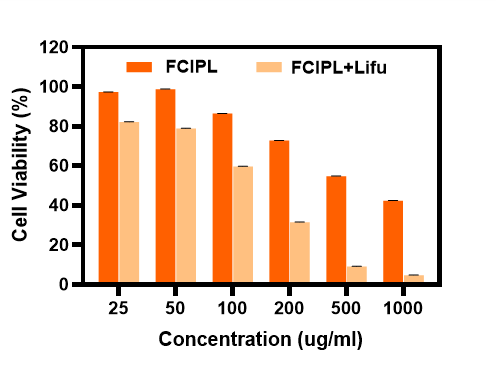


**Figure S14**. Cytotoxicity of FCIPL nanoparticles treated with or without ultrasound.


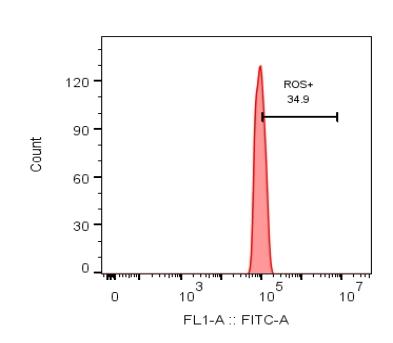

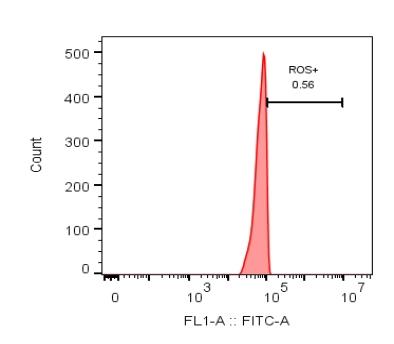

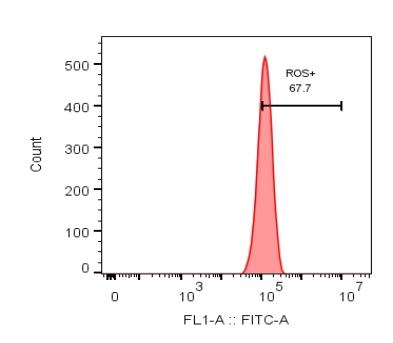

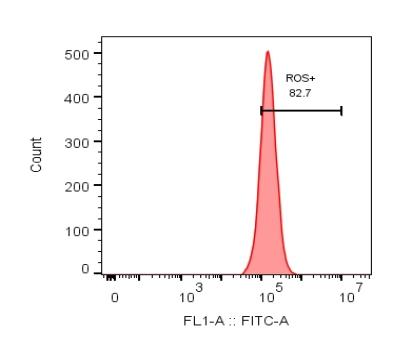

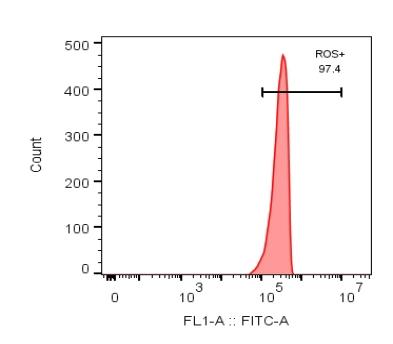

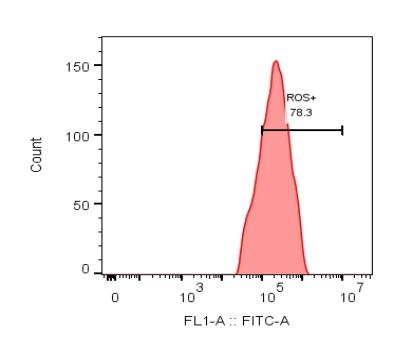


**Control**

**Lifu**

**Cur**

**FCr**

**FCIPL**

**FCIPL+Lifu**

S11

**Figure S15**. Flow cytometry was used to detect ROS content in BHT-101 cells after different treatments.


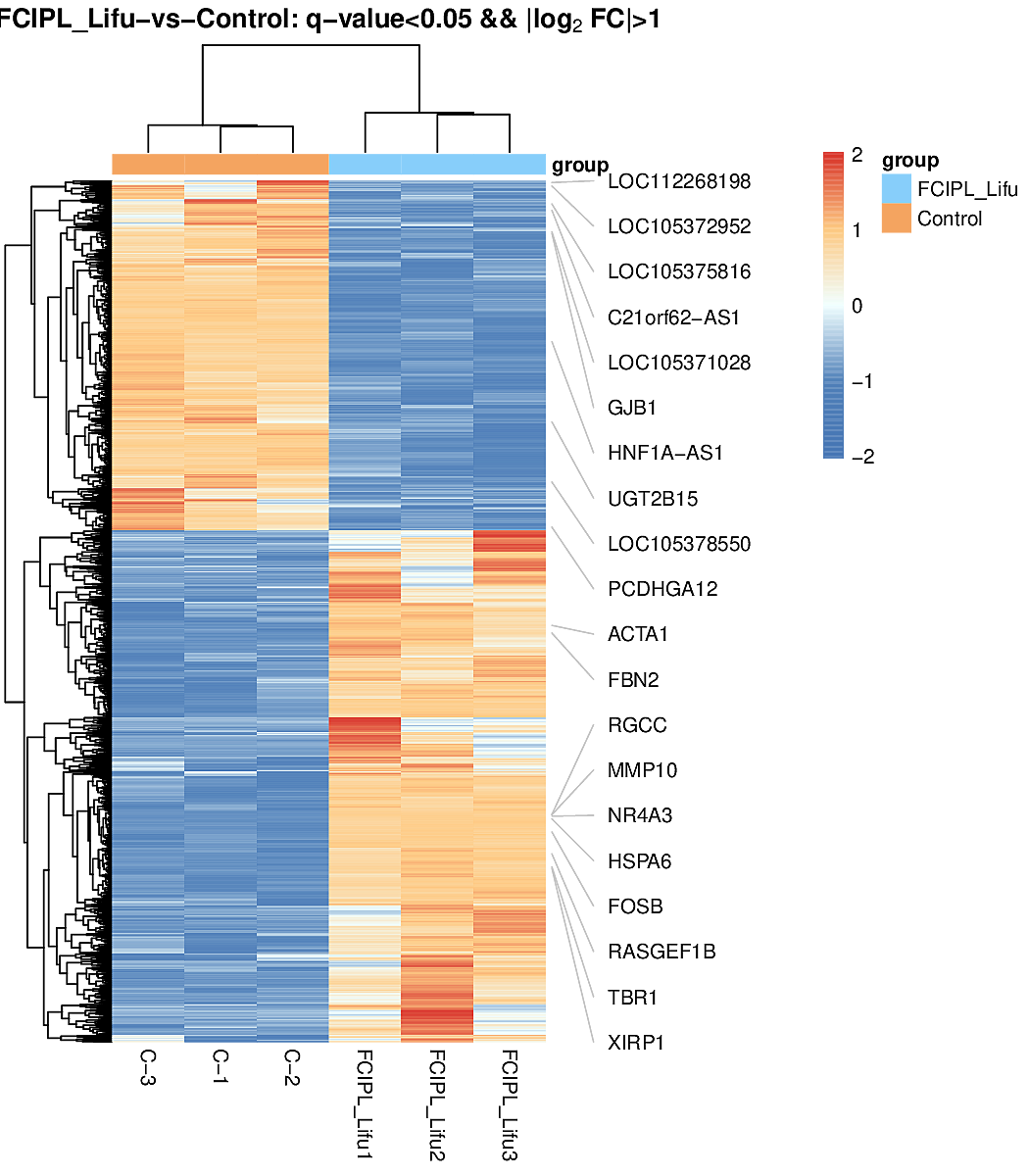


**Figure S16**. Differentially expressed genes of BHT-101 cells screened from FCIPL+Lifu and control group (with absolute fold change ≥ 1 and P < 0.05). The cluster analysis reveals significant transcriptomic differences between the control and FCIPL+Lifu groups.


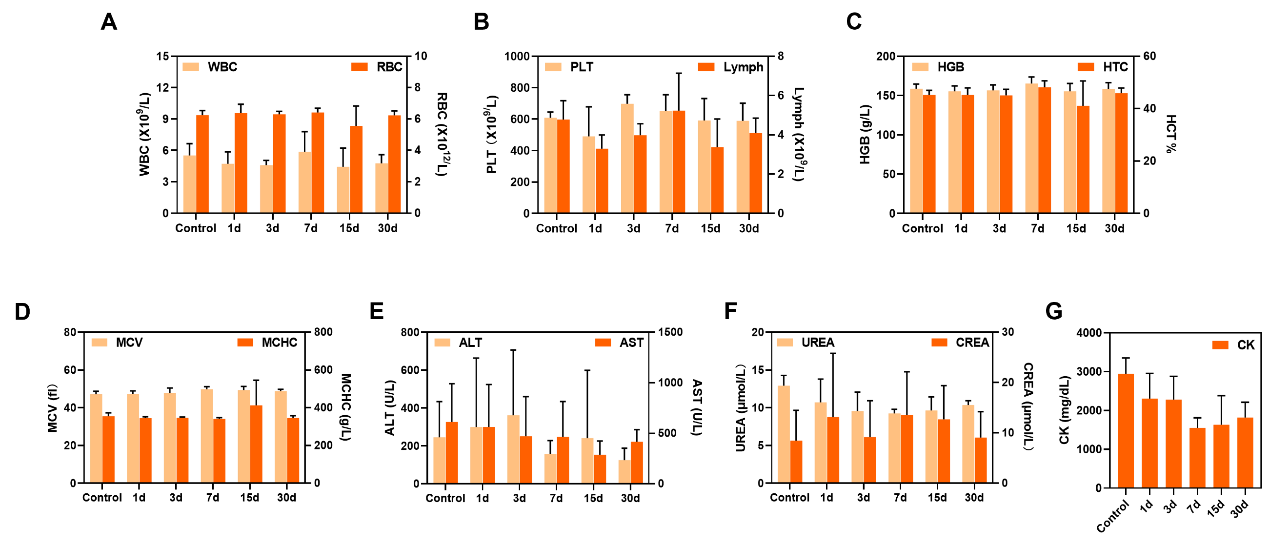


**Figure S17**. Blood routine and biochemical results of mice at 1, 3, 7, 15 and 30 days after injection of FCIPL nanoparticles


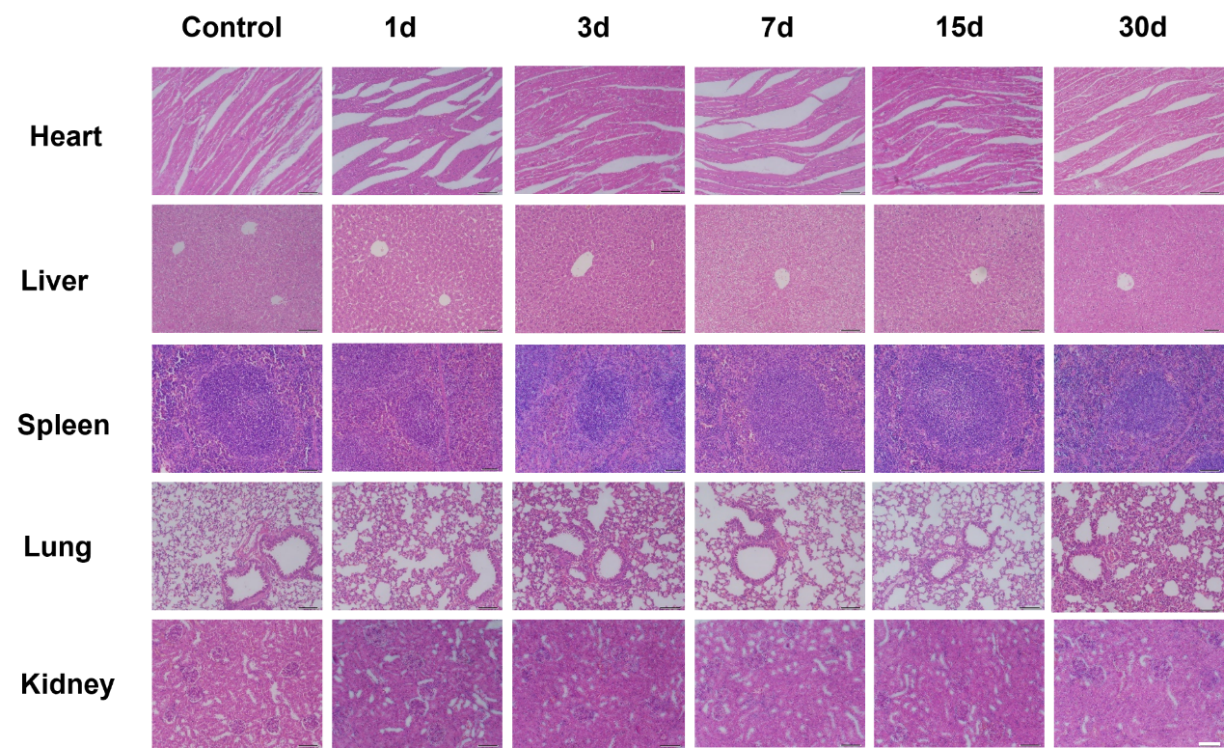


**Figure S18**. Comparison of H&E staining of vital organs of mice and control group at 1, 3, 7, 15 and 30 days after injection of FCIPL nanoparticles, the scale was 100μm.
